# Supplementary material for: Telomere-to-telomere DNA replication timing profiling using single-molecule sequencing with Nanotiming
Source: Nat Commun. 2025 Jan 2;16:242. doi: 10.1038/s41467-024-55520-3 (PMC11696806; doi:10.1038/s41467-024-55520-3)
Supplement: Supplementary file 1 — Supplementary Information [file 41467_2024_55520_MOESM1_ESM.pdf]

Telomere-to-telomere DNA replication timing profiling using single-molecule sequencing with Nanotiming

SUPPLEMENTARY INFORMATION

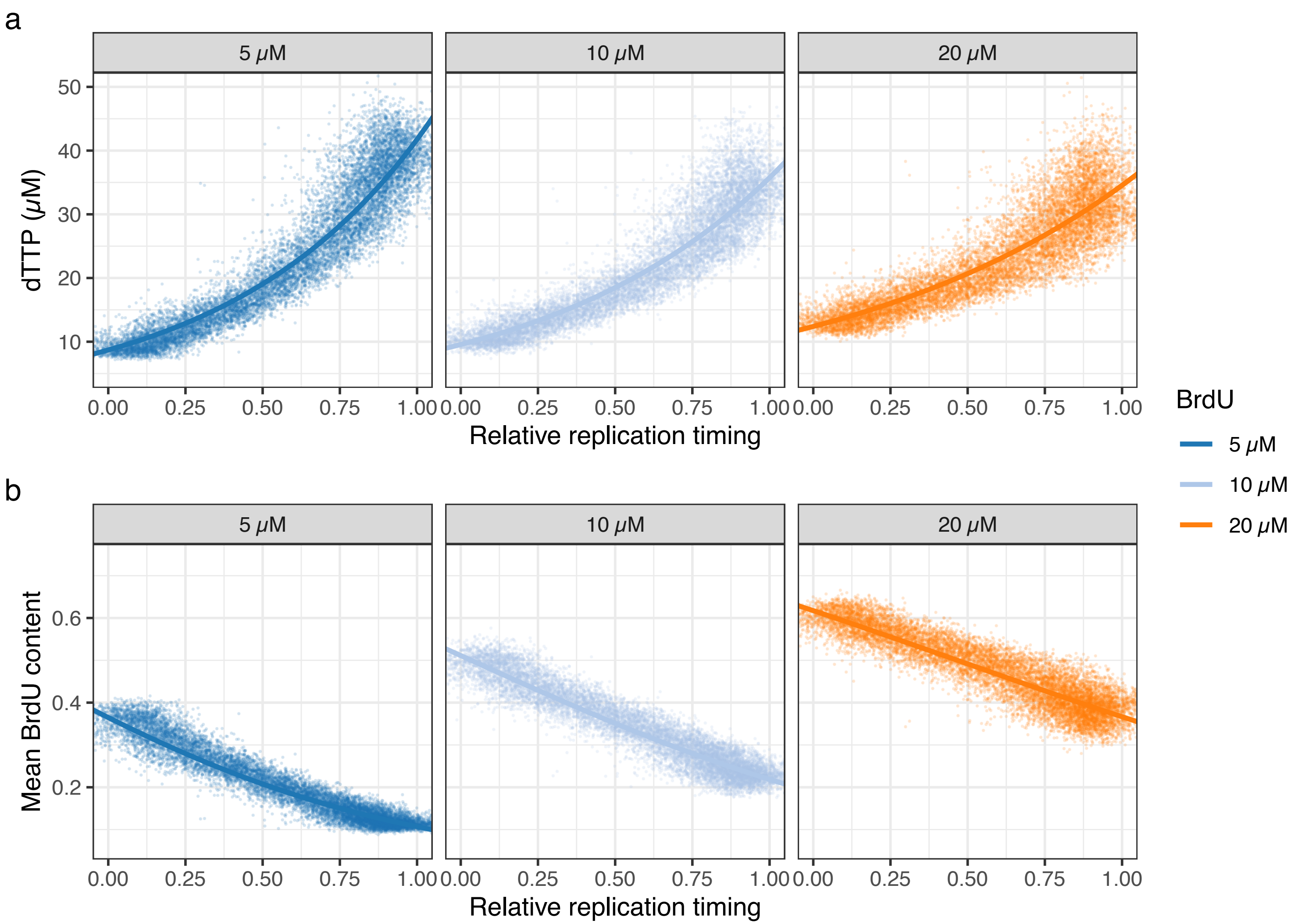

Supplementary Figure 1. Relationship between dTTP level and mean BrdU content in S phase. a, Predicted evolution of dTTP level during S phase in BT1 cells. For 5, 10 and 20 μM BrdU labelling doses, the mean BrdU content value (MBC) of every 1 kb bin of BT1 genome (y coordinate of Fig. 1c data points) was converted into a dTTP concentration (T) based on the formula T=B*(1/MBC-1), assuming that B equals the labelling BrdU concentration. The resulting T values were then plotted against the corresponding sort-seq relative copy number values (x coordinate of Fig. 1c data points) normalized between 0 and 1 corresponding to the start and end of S phase, respectively; this amounts to following dTTP level in the course of S phase. All three BrdU labelling concentrations recovered a similar, exponentially-shaped increase of dTTP during S phase. Coloured curves, exponential fits of the data. **b**, Mean BrdU content versus sort-seq relative copy number in 1 kb bins of BT1 genome as in Fig. 1c except that sort-seq data were normalized between 0 (start of S phase) and 1 (end of S phase) as in **a**. For each BrdU labelling concentration, the coloured curve was computed by implanting the exponential function determined in **a** into the MBC=B/(B+T) formula. **a**, **b**, See text and Methods for details.

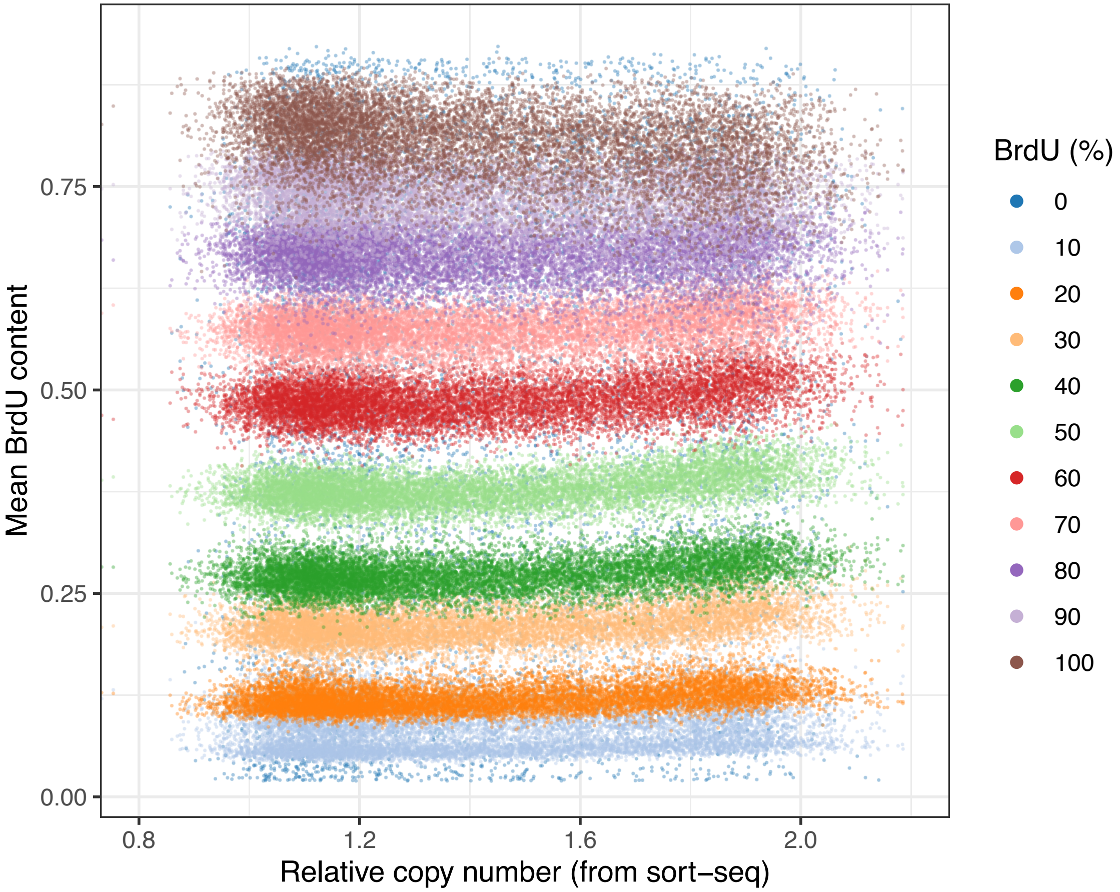

Supplementary Figure 2. Comparison between relative copy number established by sort-seq in 1 kb bins of BT1 genome and mean BrdU content in the cognate bins computed from nanopore reads of genomic DNA of MCM869 cells grown with different proportions of BrdU in the culture medium. BrdU percentages range from 0 (thymidine control) to 100% in 10% increments; MCM869 data are from ref. [13]; BT1 sort-seq data were used as a proxy for MCM869's since both strains share the same replication program[13].

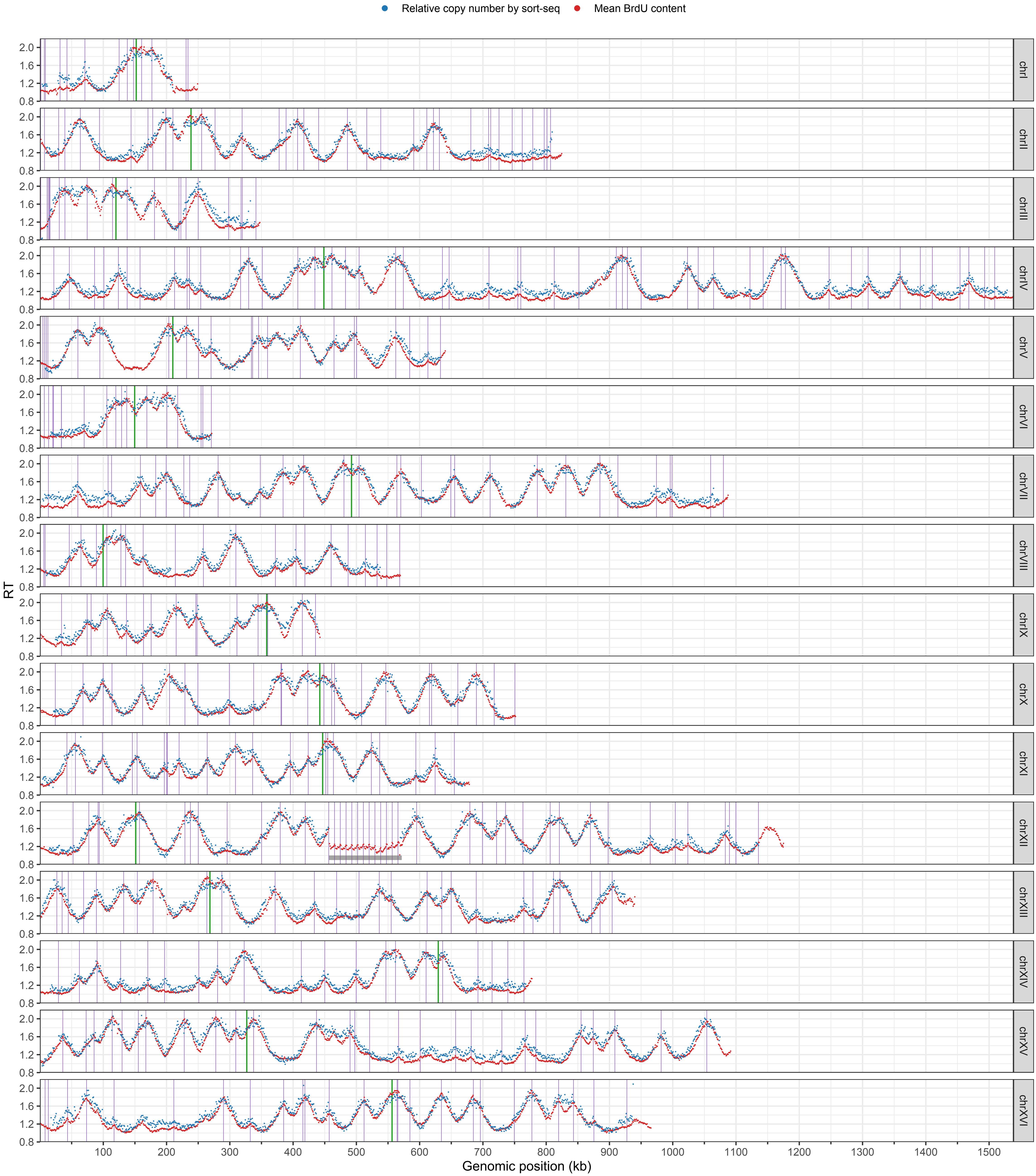

Supplementary Figure 3. Comparison between mean BrdU content and sort-seq relative copy number profiles of all yeast chromosomes. BT1 wt_rep1 mean BrdU content profile is shown. See Fig. 2 caption for details.

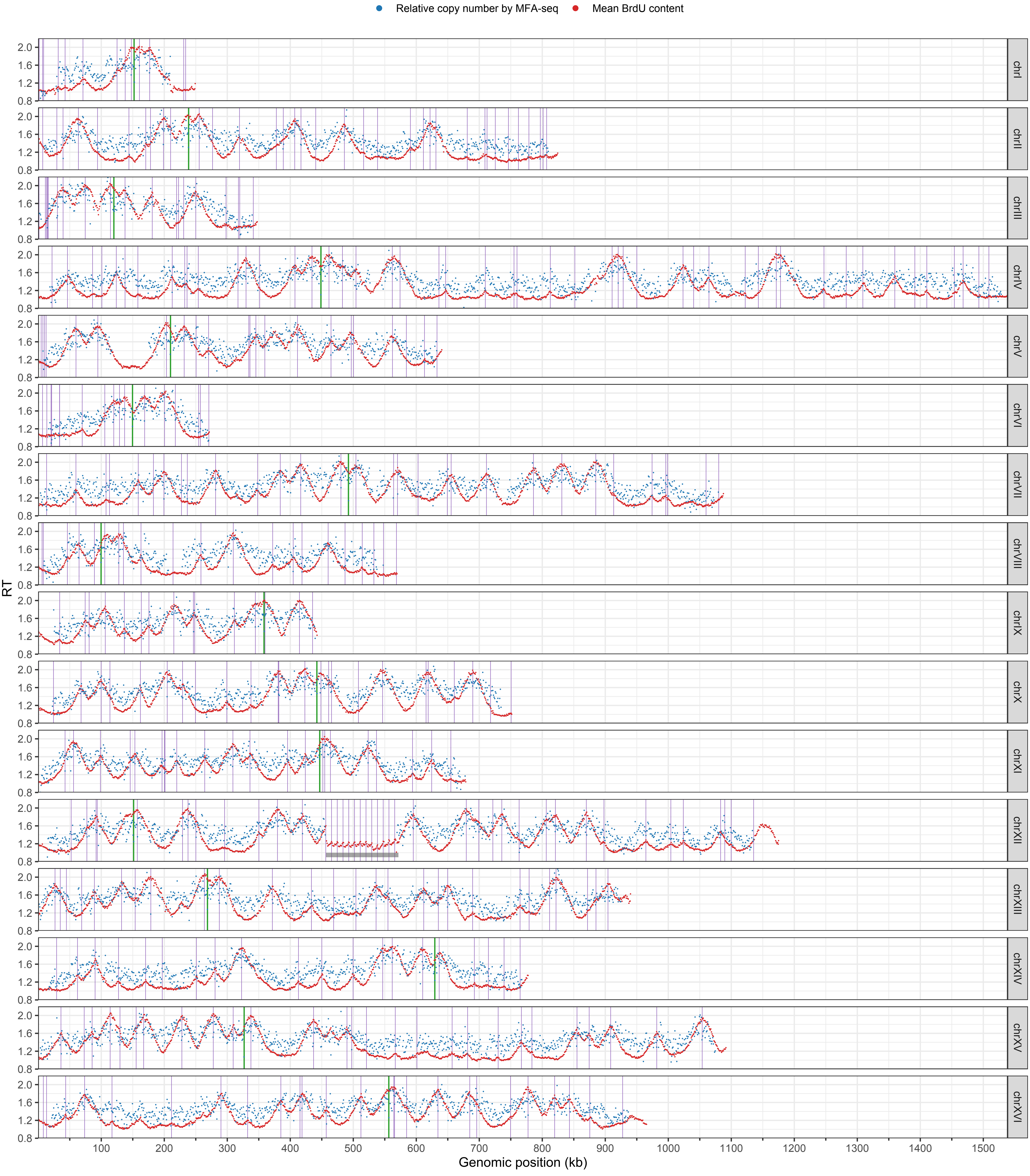

Supplementary Figure 4. Comparison between mean BrdU content and MFA-seq relative copy number profiles of all yeast chromosomes. BT1 wt_rep1 mean BrdU content profile is shown. See Fig. 2 caption for details.

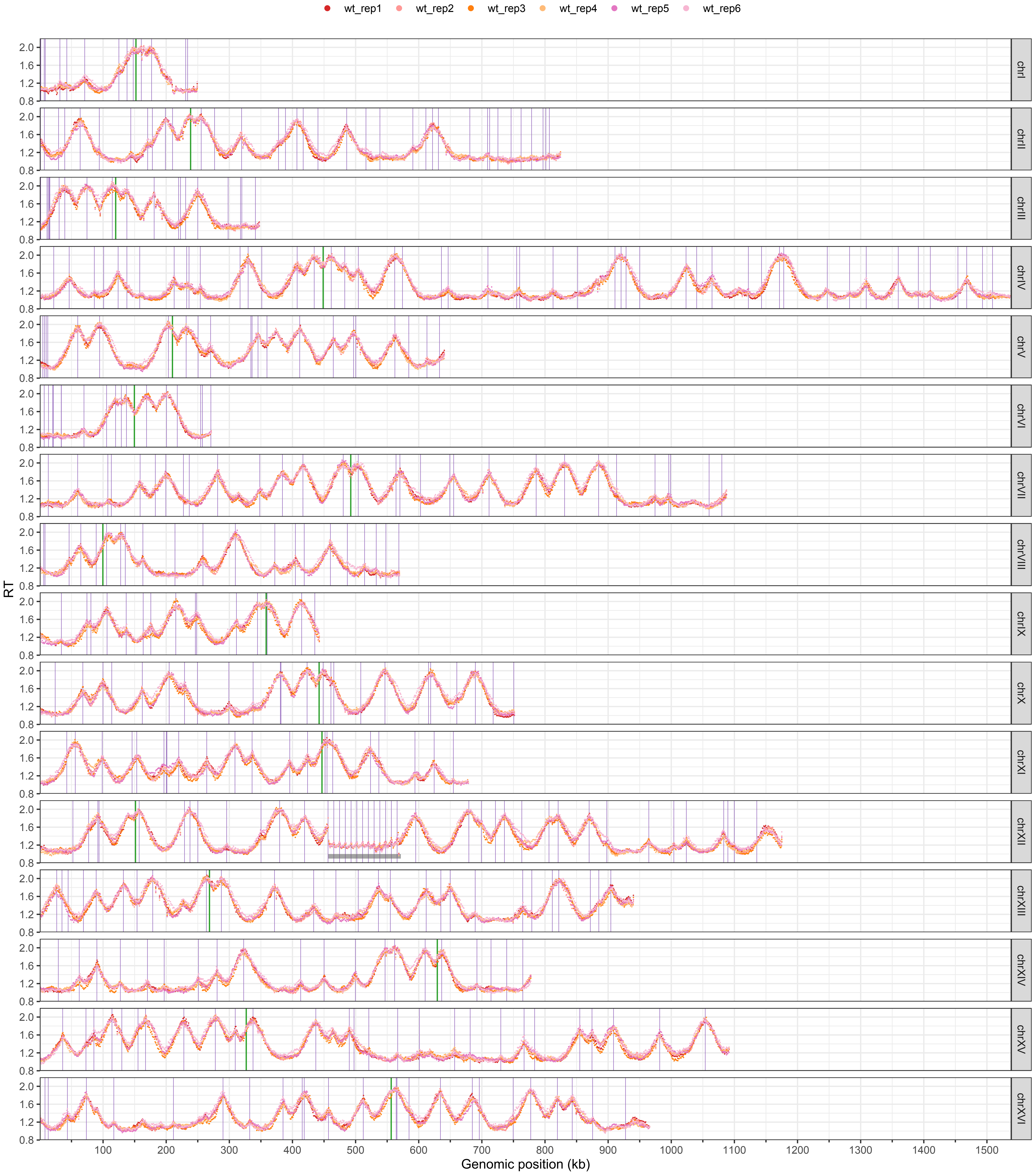

Supplementary Figure 5. Reproducibility of mean BrdU content profiling of BT1 chromosomes. Mean BrdU content profiles were computed using reads of genomic DNA from six independent BT1 cell cultures labelled for one doubling time with 5 μM BrdU. See Fig. 2 caption for details.

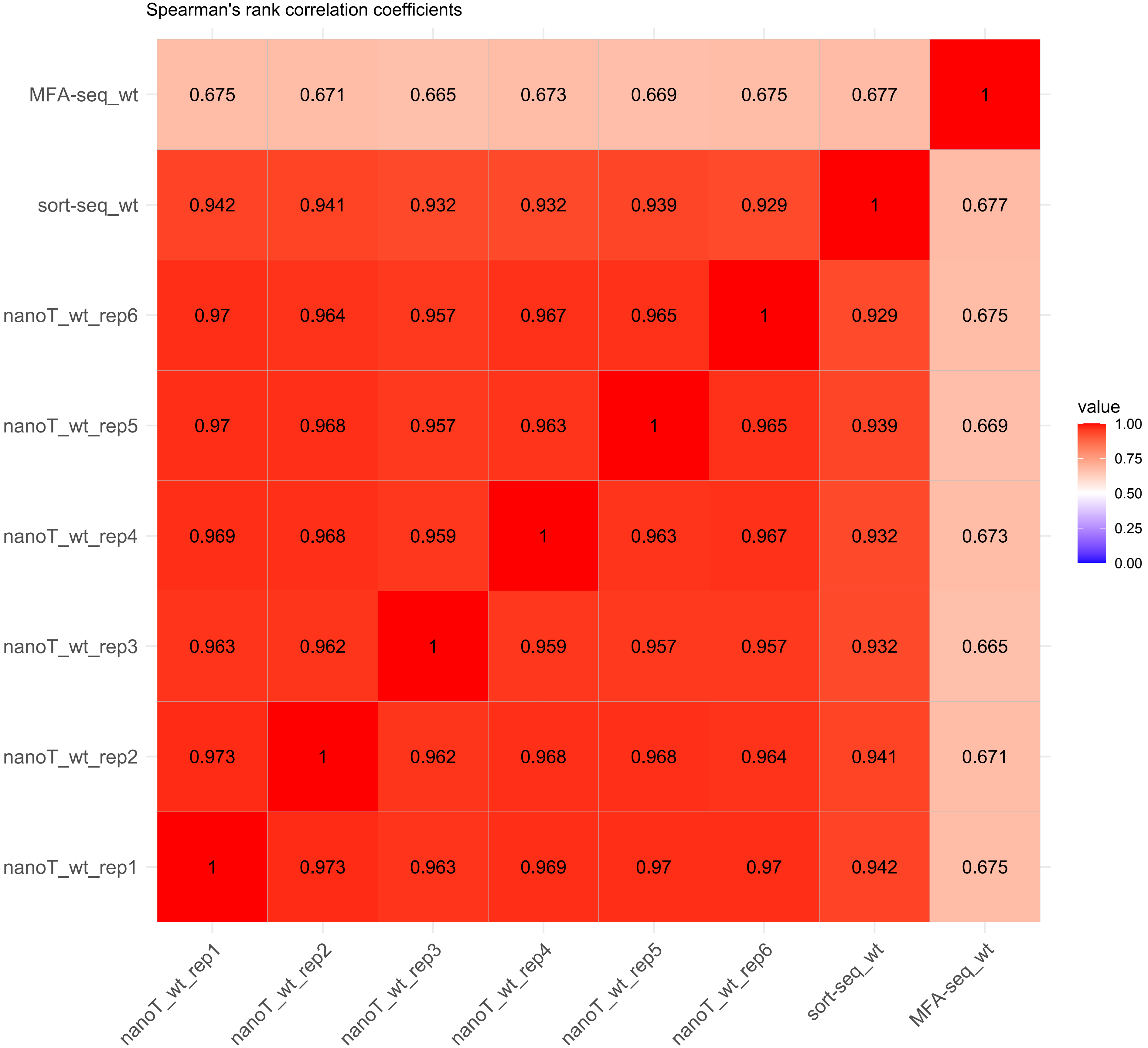

Supplementary Figure 6. Spearman's rank correlation coefficients of pairwise comparisons between six independent mean BrdU content profiles, one relative copy number profile from sort-seq and one relative copy number profile from MFA-seq of *S. cerevisiae* genome. Mean BrdU content and sort-seq profiles were computed from reads of genomic DNA of BT1 cells; MFA-seq data are from ref. [8]. nanoT, Nanotiming; wt, wild-type; rep, replicate.

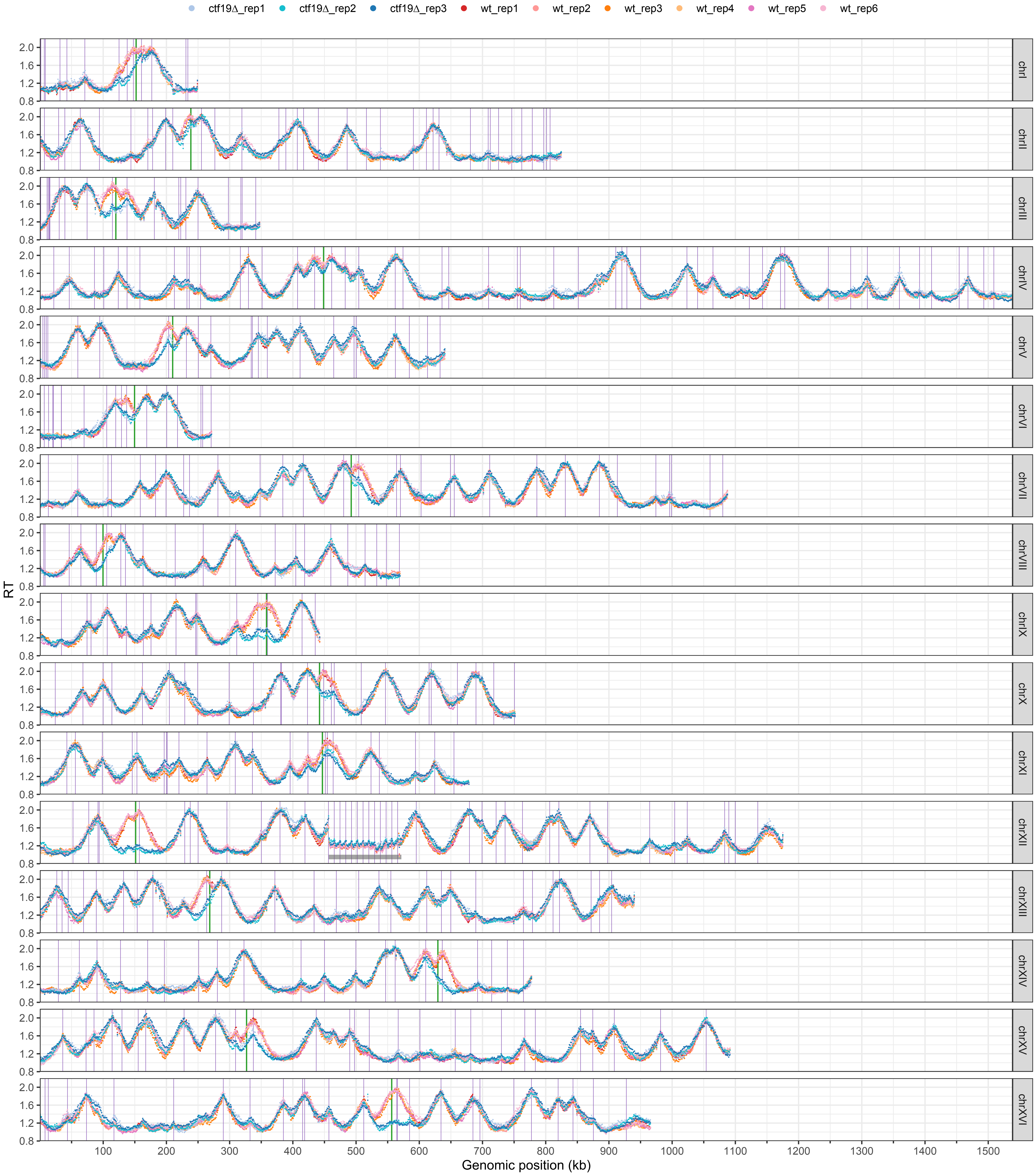

Supplementary Figure 7. Mean BrdU content profiles of all chromosomes of wild-type and *ctf19Δ* BT1 cells. See Fig. 2 caption for details.

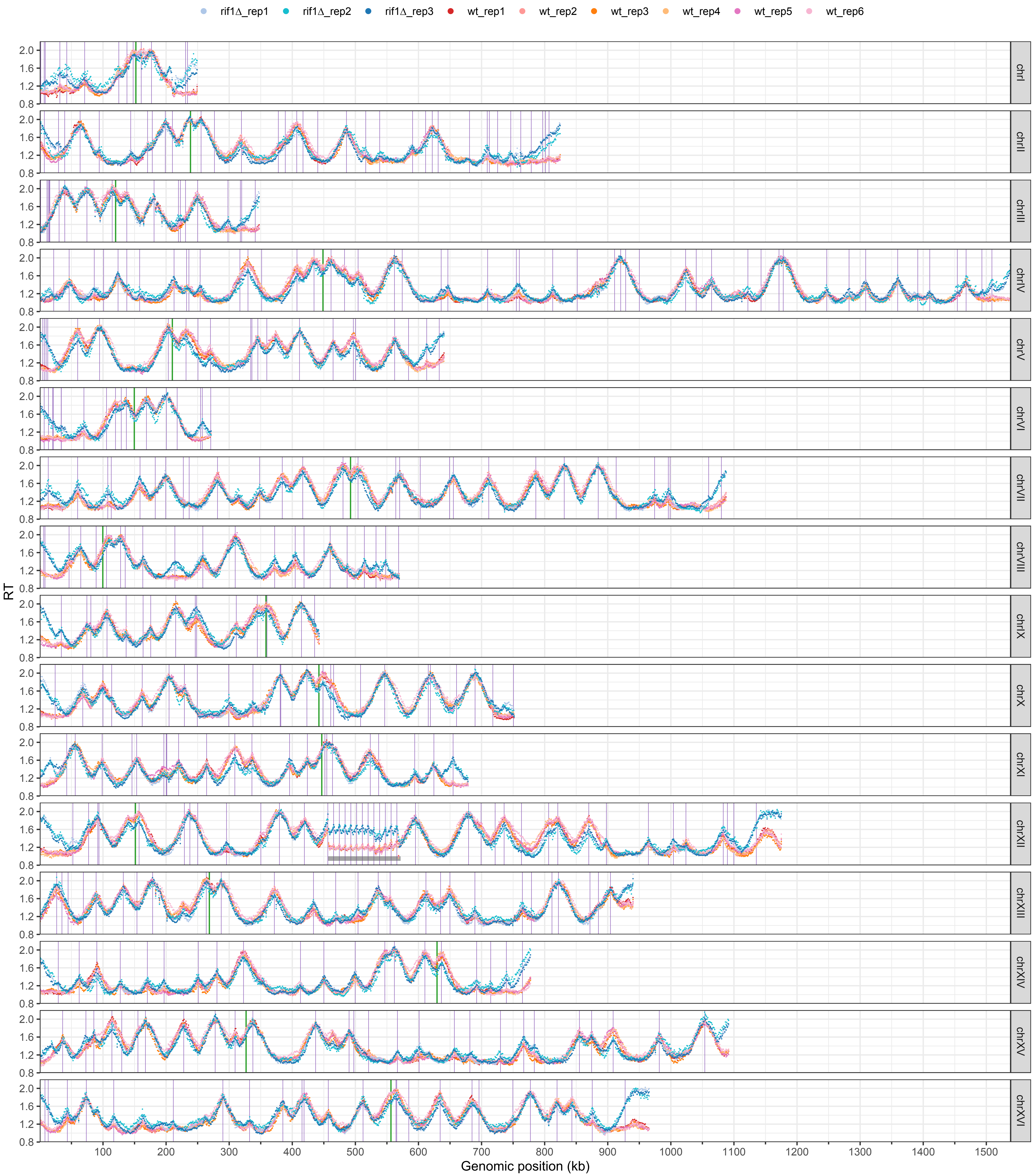

Supplementary Figure 8. Mean BrdU content profiles of all chromosomes of wild-type and *rif1Δ* BT1 cells. See Fig. 2 caption for details.

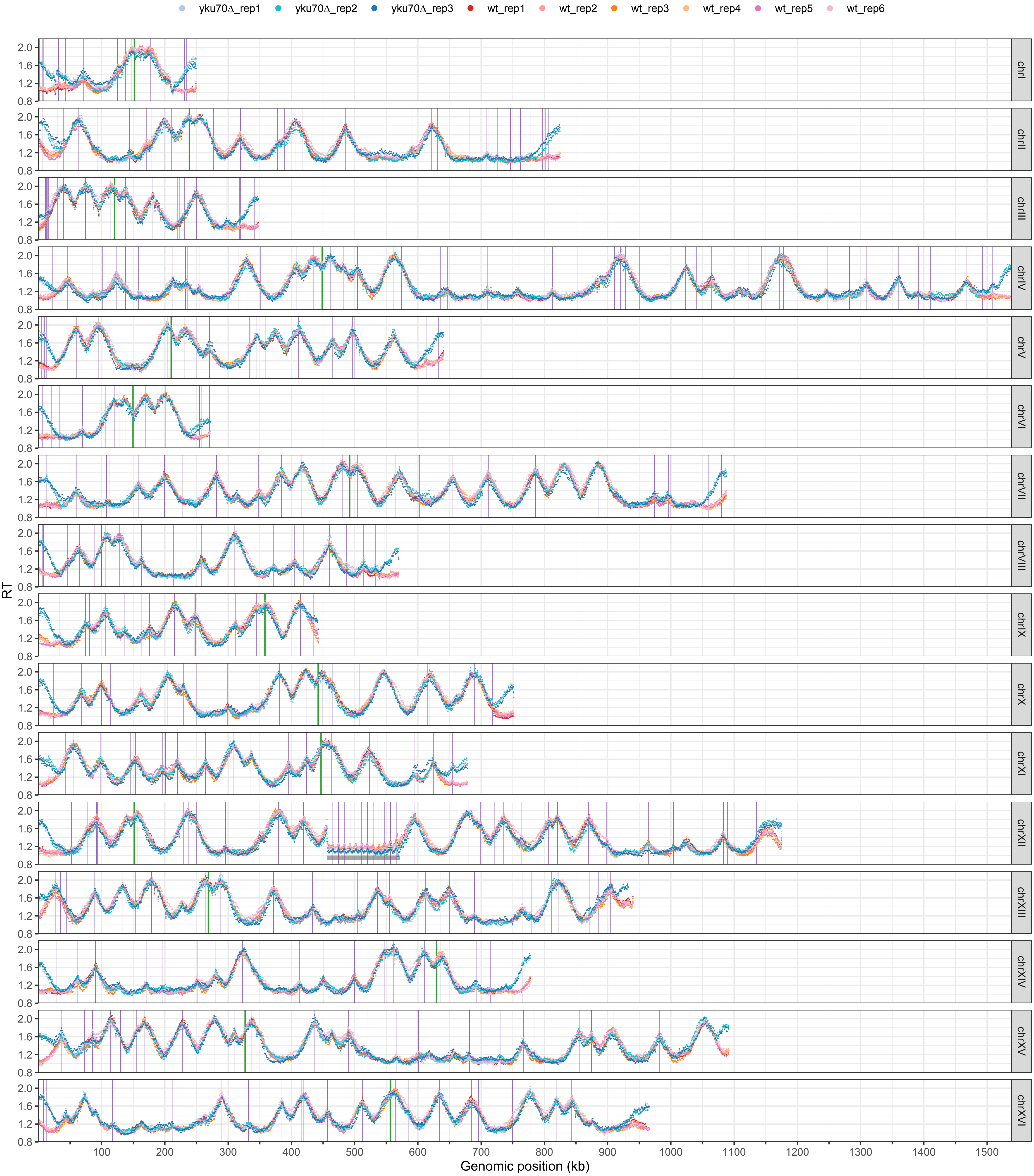

Supplementary Figure 9. Mean BrdU content profiles of all chromosomes of wild-type and *yku70Δ* BT1 cells. See Fig. 2 caption for details.

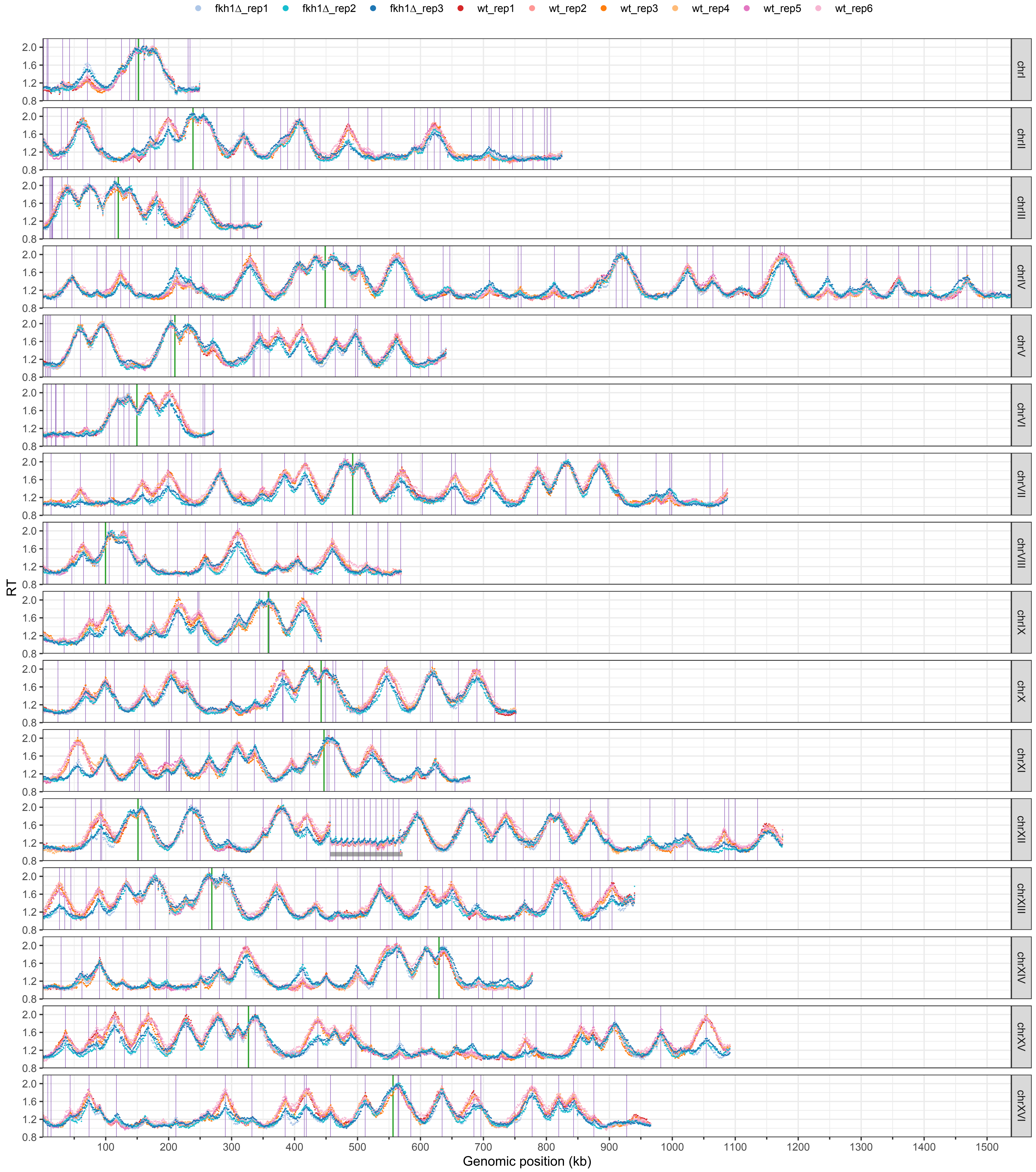

Supplementary Figure 10. Mean BrdU content profiles of all chromosomes of wild-type and *fkh1Δ* BT1 cells. See Fig. 2 caption for details.

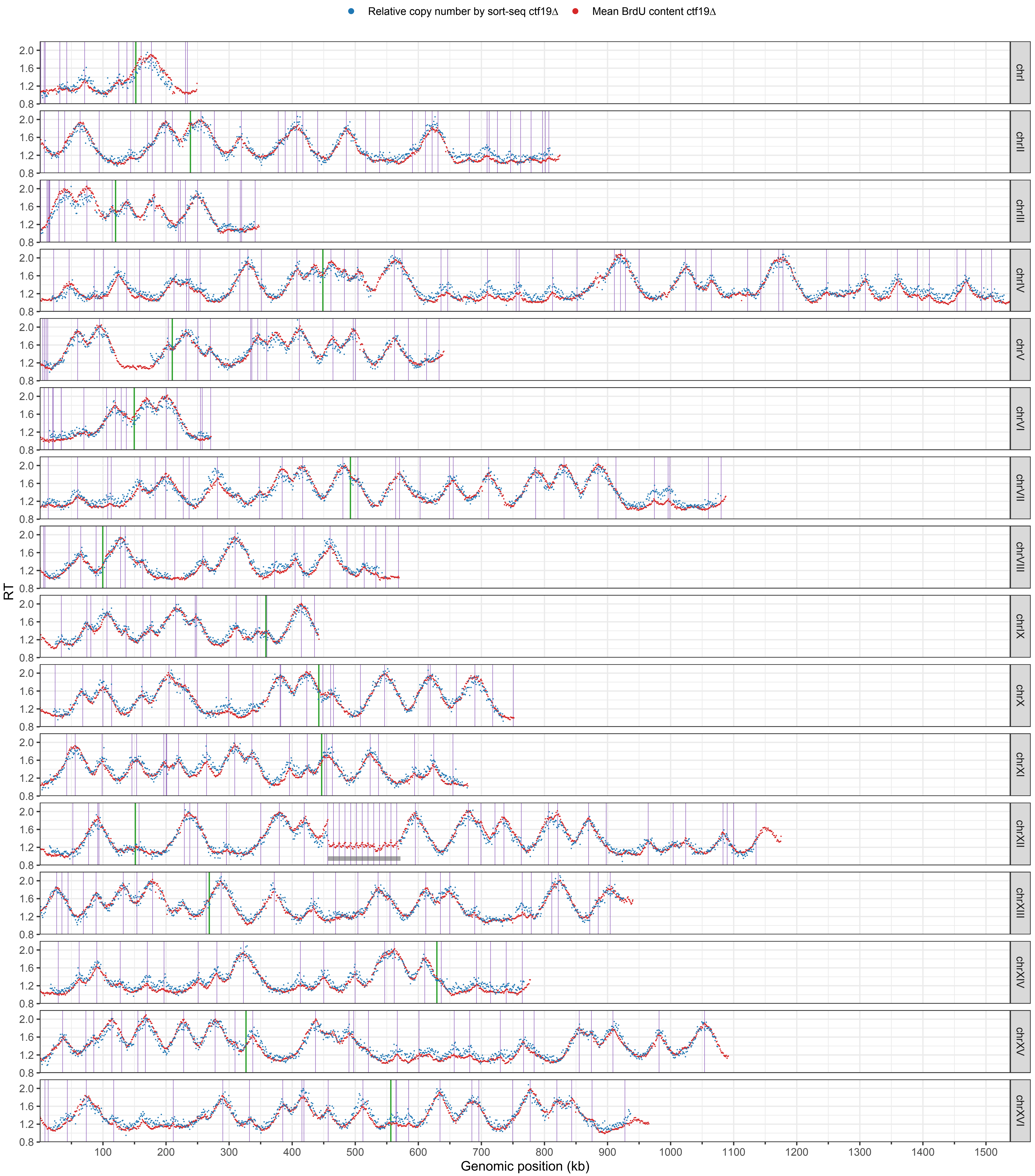

Supplementary Figure 11. Comparison between mean BrdU content and sort-seq relative copy number profiles of all chromosomes of *S. cerevisiae ctf19Δ* cells. Mean BrdU content profile was computed from reads of genomic DNA of *ctf19Δ* BT1 cells (rep3); sort-seq data are from ref. [25]. See Fig. 2 caption for details.

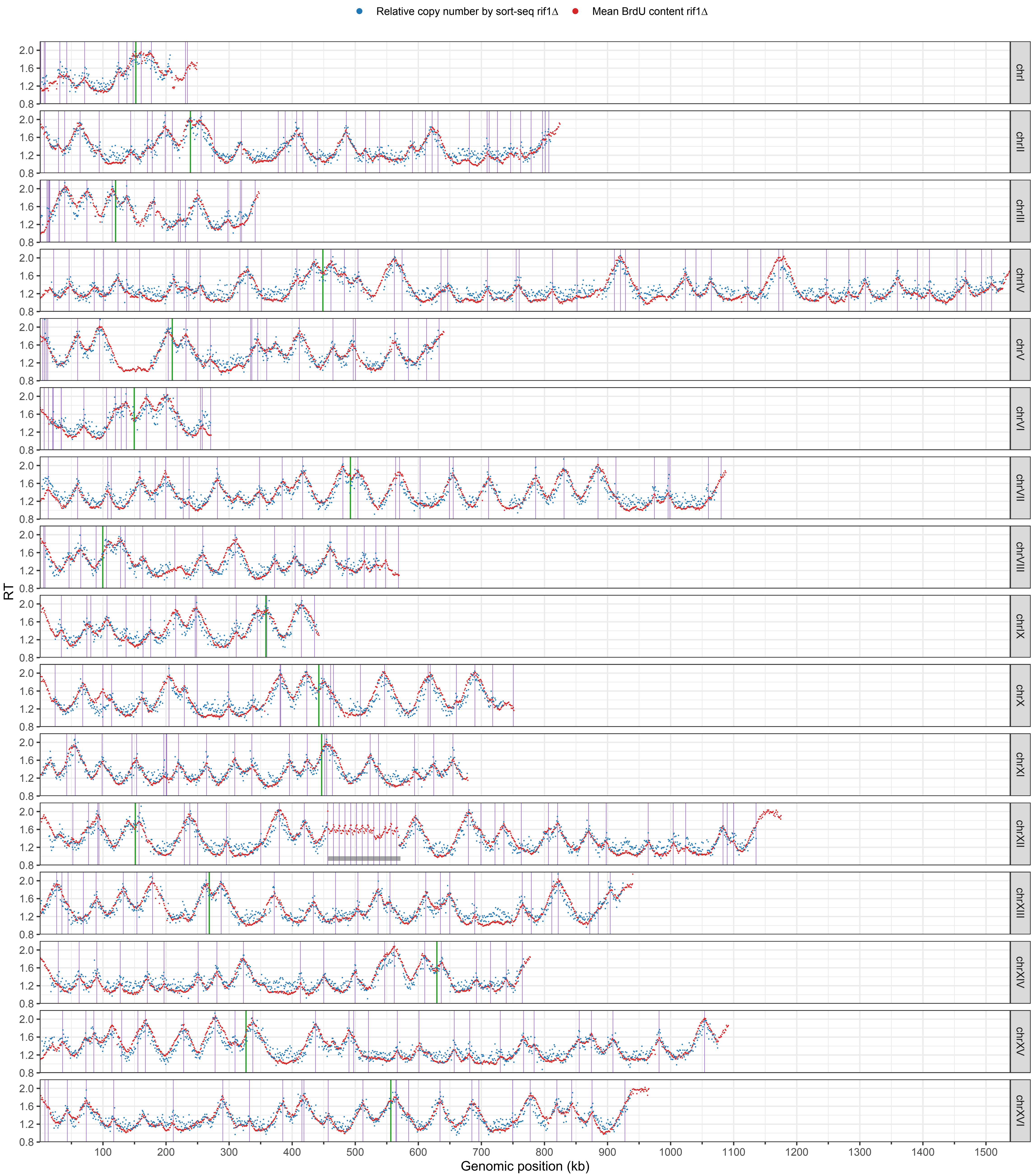

Supplementary Figure 12. Comparison between mean BrdU content and sort-seq relative copy number profiles of all chromosomes of *S. cerevisiae rif1Δ* cells. Mean BrdU content profile was computed from reads of genomic DNA of *rif1Δ* BT1 cells (rep1); sort-seq data are from ref. [7]. See Fig. 2 caption for details.

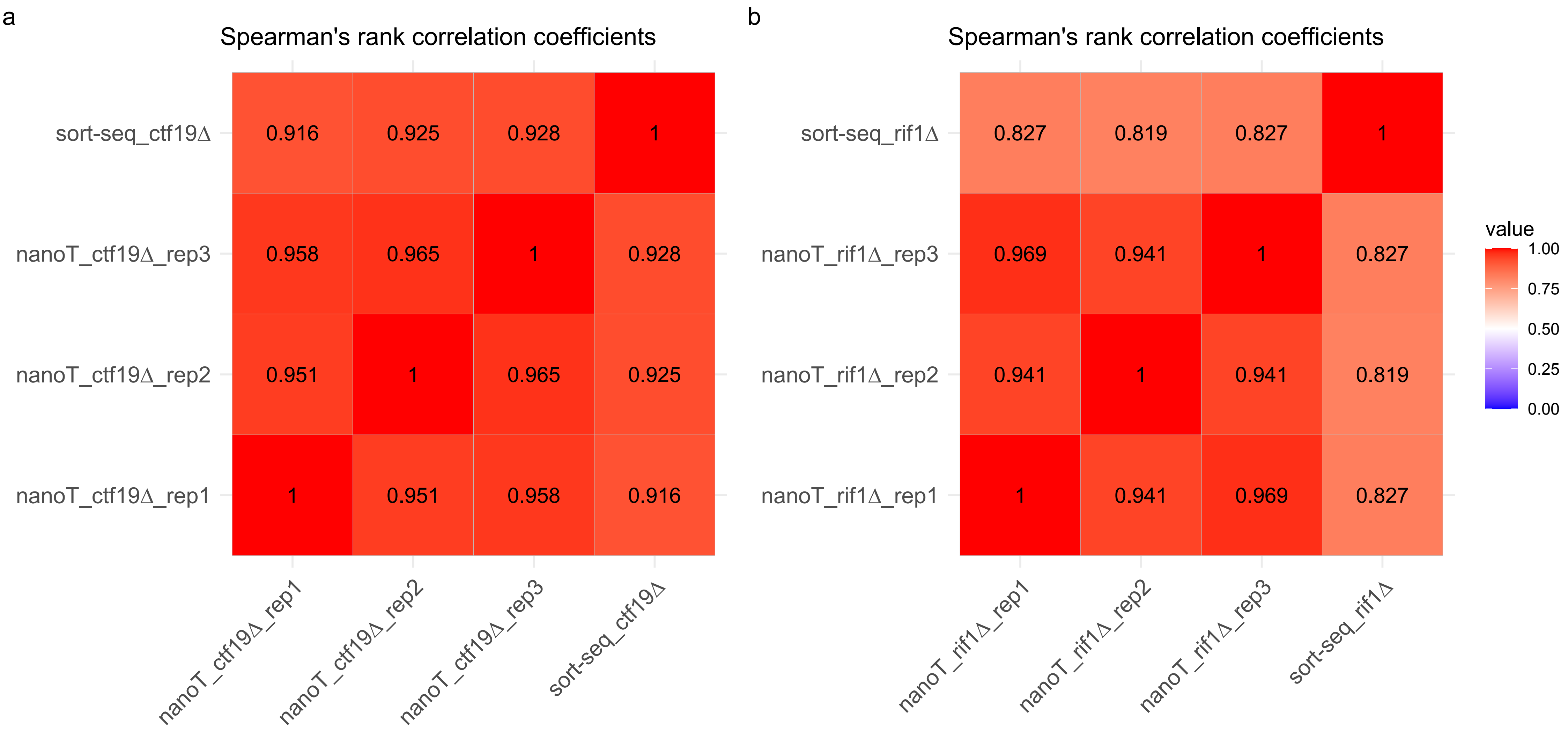

Supplementary Figure 13. Spearman's rank correlation coefficients of pairwise comparisons between three independent mean BrdU content profiles and one relative copy number profile by sort-seq of *ctf19Δ* and *rif1Δ* genomes. a, **b**, Results for *ctf19Δ* (**a**) and *rif1Δ* (**b**) strains. Mean BrdU content profiles were computed from reads of genomic DNA of *ctf19Δ* (**a**) and *rif1Δ* (**b**) BT1 cells; *ctf19Δ* and *rif1Δ* sort-seq data are from ref. [25] and ref. [7], respectively. nanoT, Nanotiming; rep, replicate.

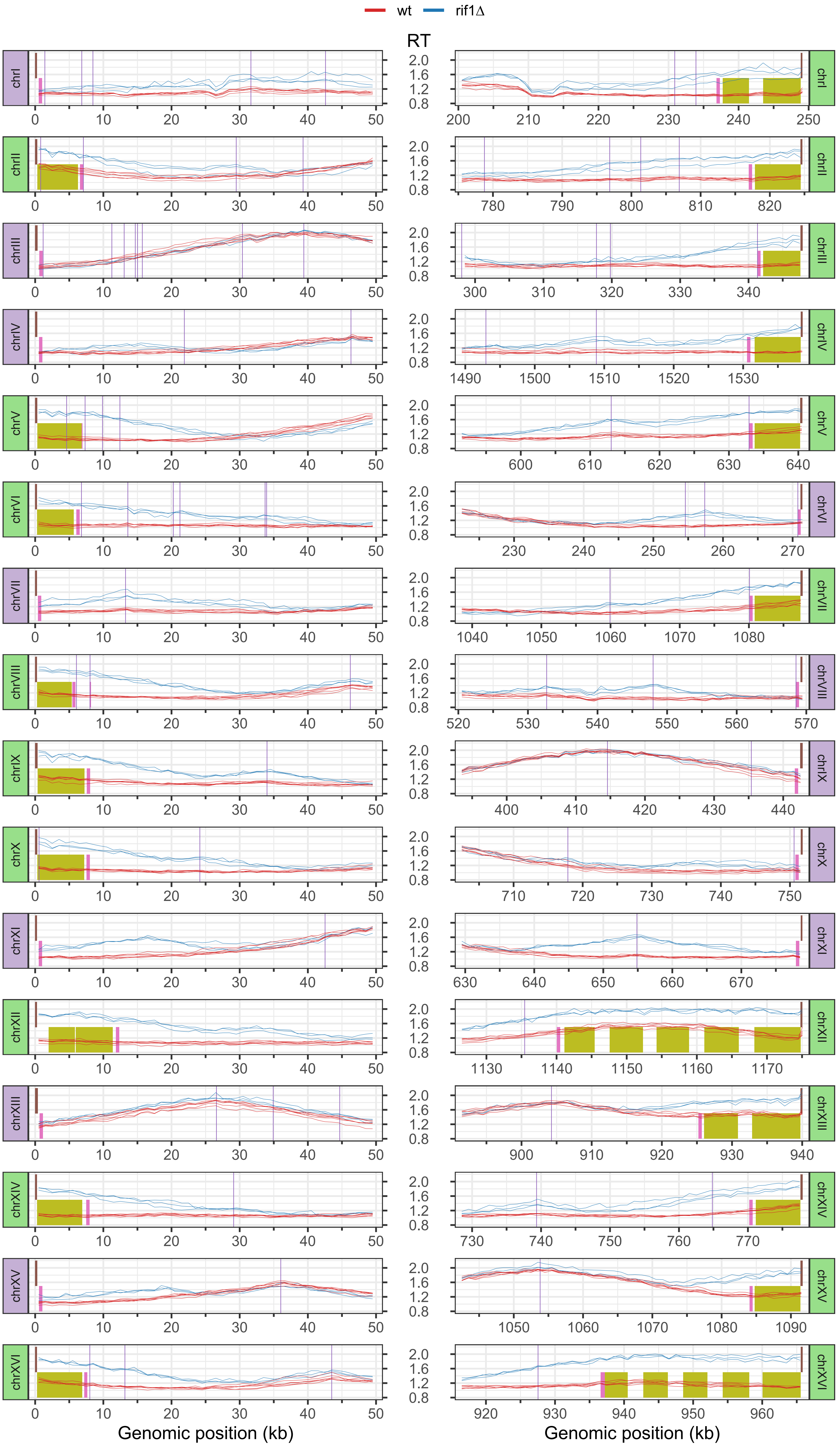

Supplementary Figure 14. Mean BrdU content profiles over 50 kb of the left and right extremities of *S. cerevisiae* chromosomes in wild-type and *rif1Δ* BT1 cells. See Fig. 3 caption for details.

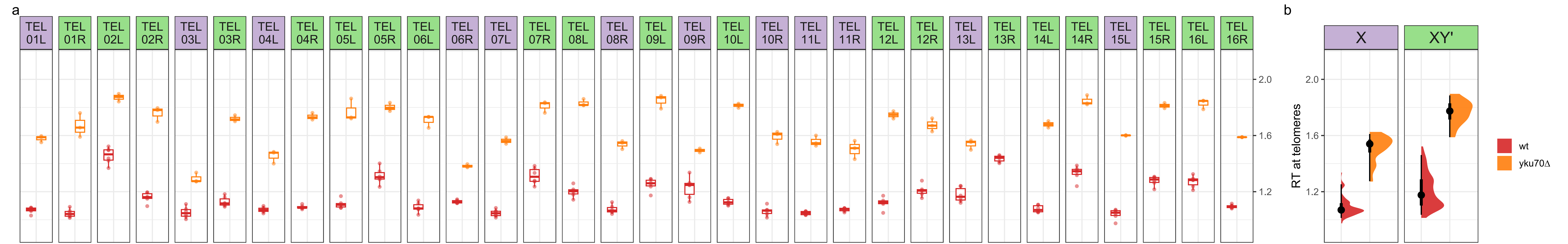

Supplementary Figure 15. Yku70 regulates the RT of *S. cerevisiae* X and XY' telomeres. a, RT at individual telomeres in wild-type (wt) and *yku70Δ* BT1 cells. $n_{wt} = 6$ mean BrdU content values from independent cell cultures, $n_{yku70\Delta} = 3$. **b**, RT at X and XY' telomeres in wt and *yku70Δ* cells. For X telomeres, $n_{wt} = 72$ mean BrdU content values, $n_{yku70\Delta} = 36$; for XY' telomeres, $n_{wt} = 120$, $n_{yku70\Delta} = 57$. **a**, **b**, wt and *yku70Δ* samples are in red and orange colours, respectively. TEL13R RT was not determined in *yku70Δ* mutant because of a missing Y' element at the right end of chromosome XIII compared to BT1 assembly, preventing proper read mapping and telomeric RT computation. See Fig. 3 caption for details.

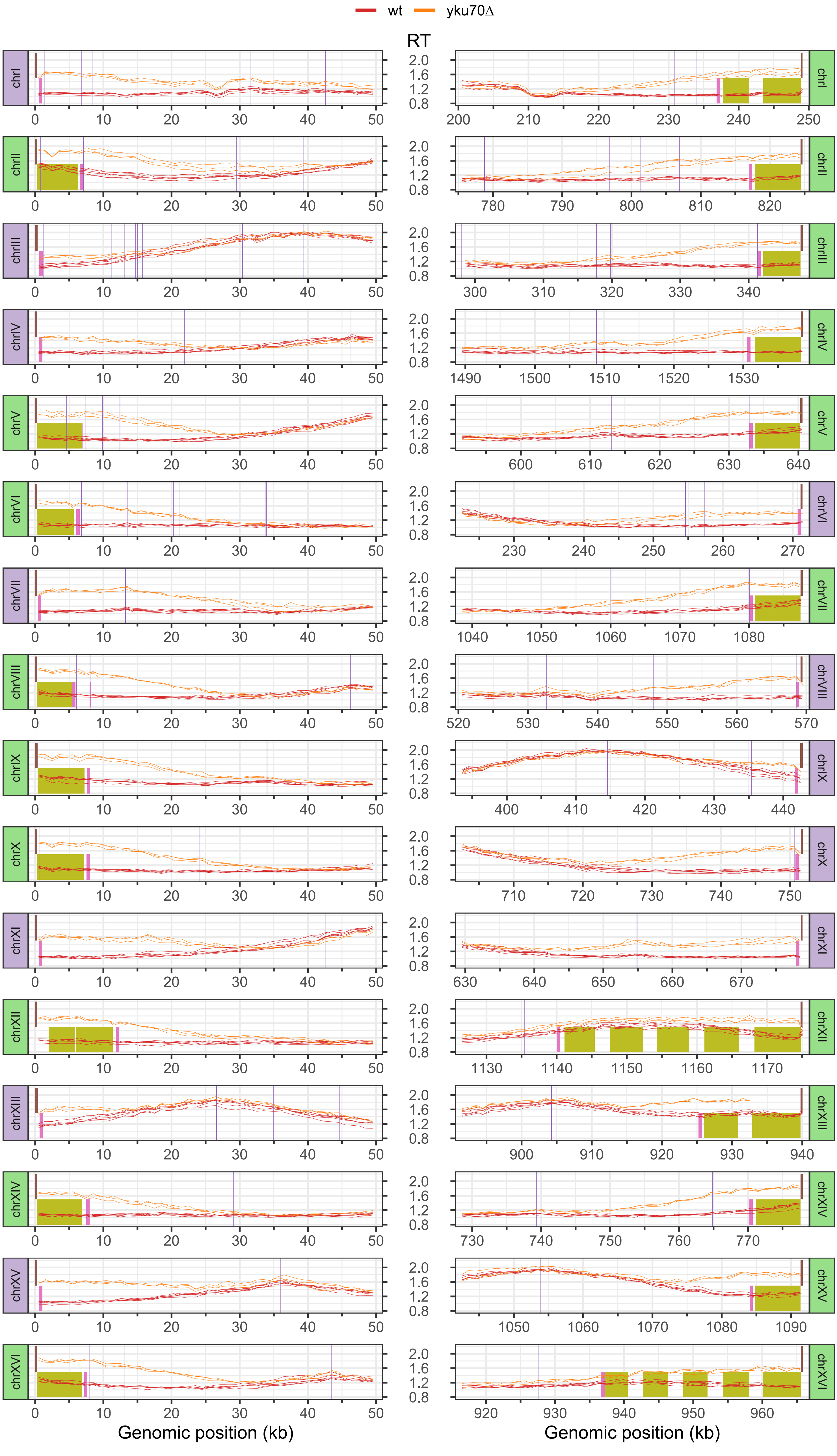

Supplementary Figure 16. Mean BrdU content profiles over 50 kb of the left and right extremities of *S. cerevisiae* chromosomes in wild-type and *yku70Δ* BT1 cells. Six and three biological replicates, corresponding to independent cell cultures, are presented for wild-type and *yku70Δ* BT1 cells, respectively. Please note the missing distal Y' element at the right end of chromosome XIII in *yku70Δ* mutant. See Fig. 3 caption for details.

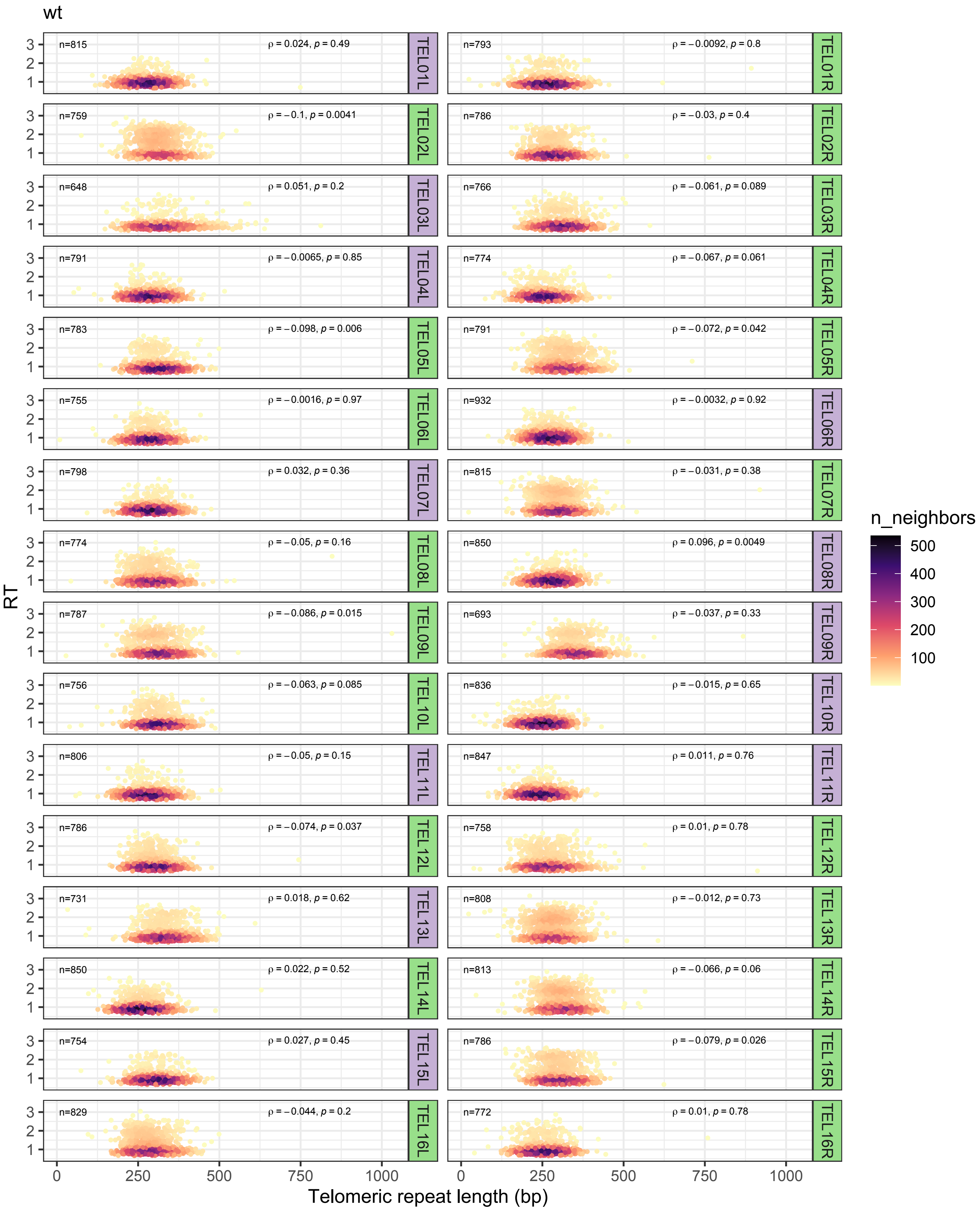

Supplementary Figure 17. Analysis of the relationship between telomere length and RT at the single-telomere level in wild-type BT1 cells. See Fig. 4 caption for details.

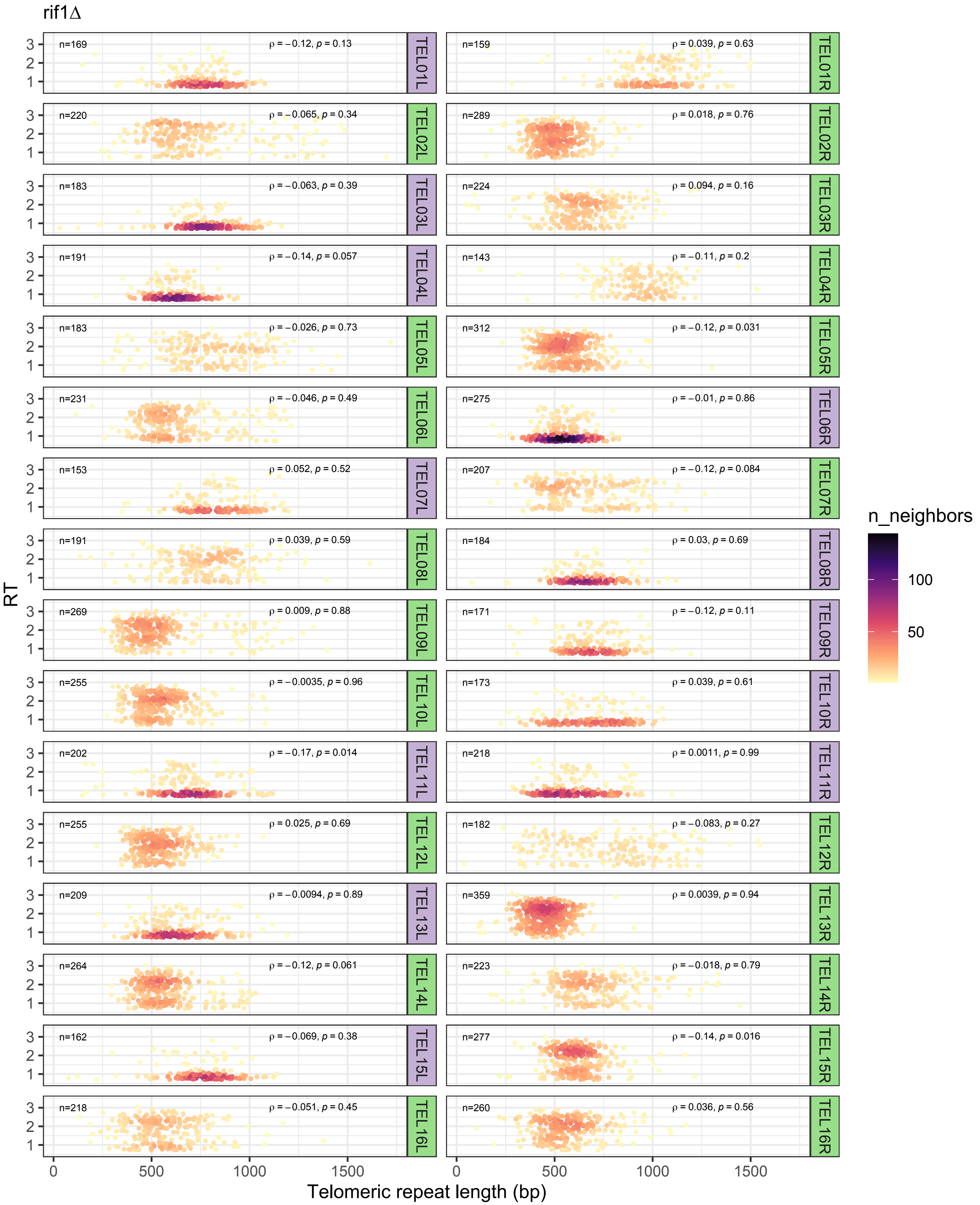

Supplementary Figure 18. Analysis of the relationship between telomere length and RT at the single-telomere level in *rif1Δ* BT1 cells. See Fig. 4 caption for details.

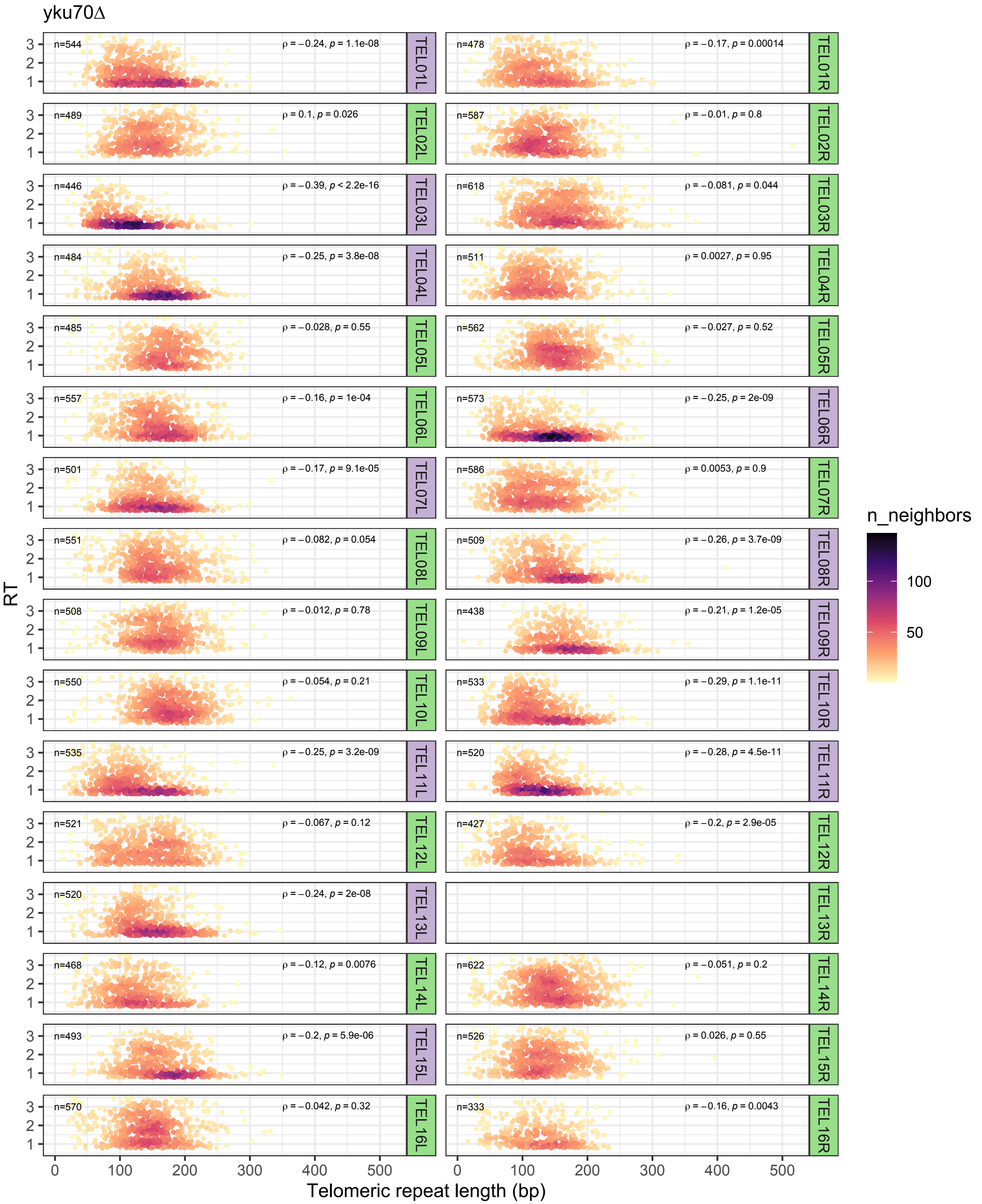

Supplementary Figure 19. Analysis of the relationship between telomere length and RT at the single-telomere level in *yku70Δ* BT1 cells. No data was computed at TEL13R because of a missing Y' element at the right end of chromosome XIII compared to BT1 assembly. See Fig. 4 caption for details.

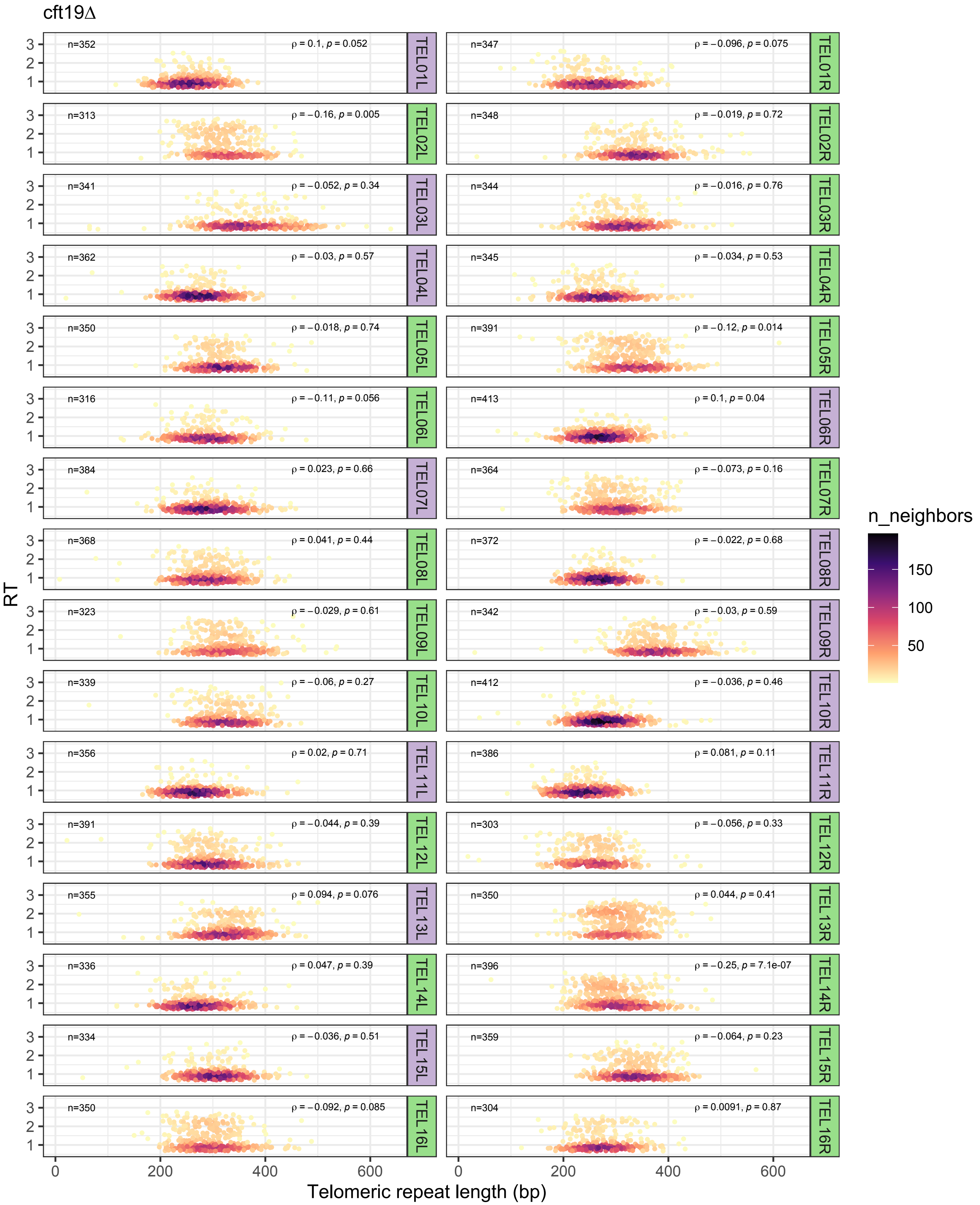

Supplementary Figure 20. Analysis of the relationship between telomere length and RT at the single-telomere level in *ctf19Δ* BT1 cells. See Fig. 4 caption for details.

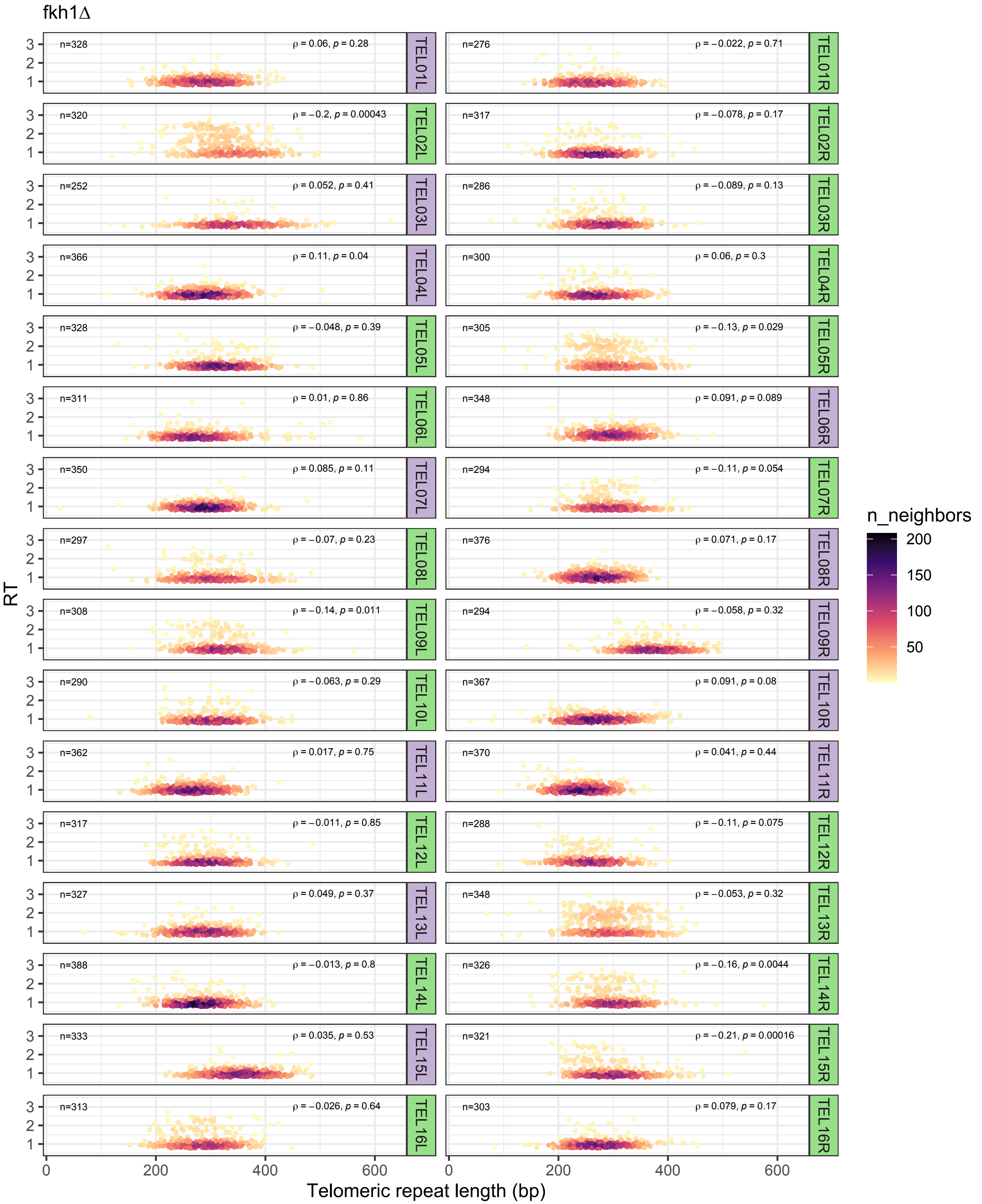

Supplementary Figure 21. Analysis of the relationship between telomere length and RT at the single-telomere level in *fkh1Δ* BT1 cells. See Fig. 4 caption for details.

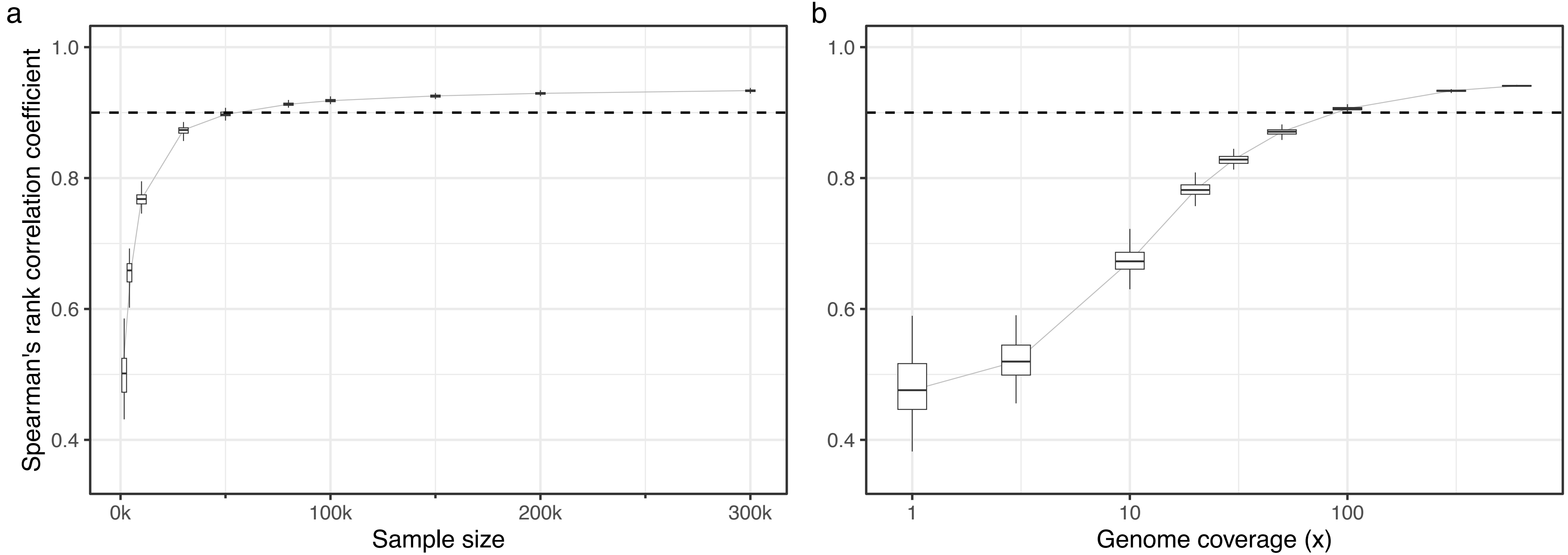

Supplementary Figure 22. Impact of the amount of sequencing data on Nanotiming RT profile accuracy. a, **b**, Evolution of Spearman's rank correlation coefficient between mean BrdU content and sort-seq profiles of BT1 genome in wild-type cells as a function either of the number of nanopore reads used to compute mean BrdU content profiles (**a**) or of genomic coverage (**b**). Horizontal dashed line corresponds to a Spearman's rank correlation coefficient of 0.9. Reads were randomly selected from BT1 wt_rep1 dataset; subsampling was performed 100 times for each read number or genomic coverage category. Horizontal black line, median; boxes, 25th to 75th percentiles; whiskers, 1.5x interquartile range. x, fold.

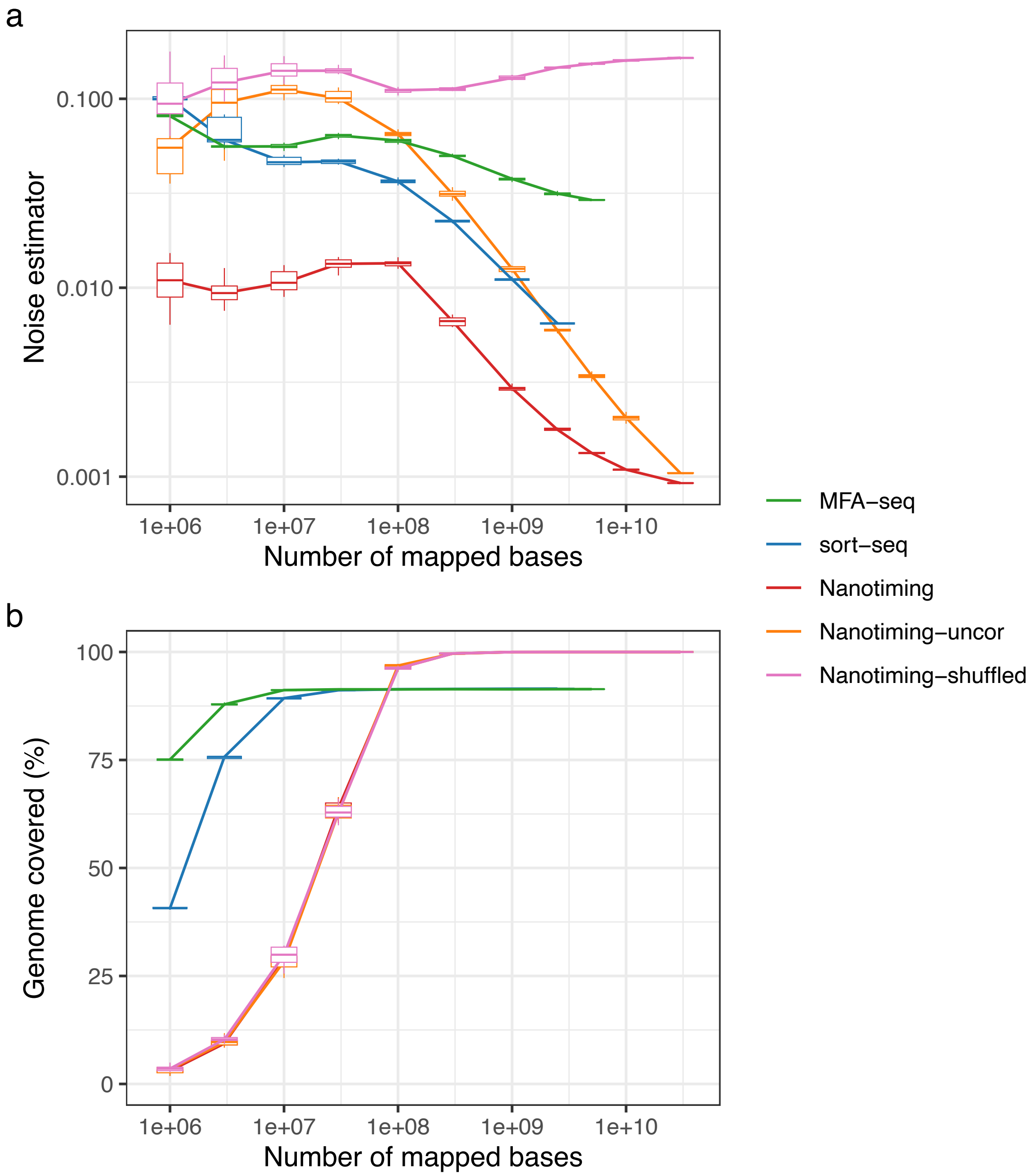

Supplementary Figure 23. Evaluation of Nanotiming, sort-seq and MFA-seq noise as a function of the number of mapped bases. a, Noise estimator corresponds to the variance of signal differences between consecutive 1 kb bins. Reads were randomly selected from the complete BT1 PromethION or sort-seq datasets or from MFA-seq data from ref. [8]; subsampling was performed 10 times for each category of number of mapped bases. Since nanopore reads are typically 10 to 20 kb in length and therefore span successive 1 kb bins, which makes neighbouring bin values not independent and may artificially reduce noise measurement, signal variation for Nanotiming was compared either between consecutive bins from the same sampling, which preserves the bin-to-bin dependency in nanopore reads (Nanotiming curve, in red) or from independent samplings, which removes this dependency (Nanotiming-uncor curve, in orange). Noise in a random RT profile was estimated using shuffled Nanotiming data (Nanotiming-shuffled, in pink) for comparison. See Methods. **b**, Percentage of *S. cerevisiae* genome covered by subsampled reads for each category of number of mapped bases. **a**, **b**, Horizontal line, median; boxes, 25th to 75th percentiles; whiskers, 1.5x interquartile range. Uncor, uncorrelated.

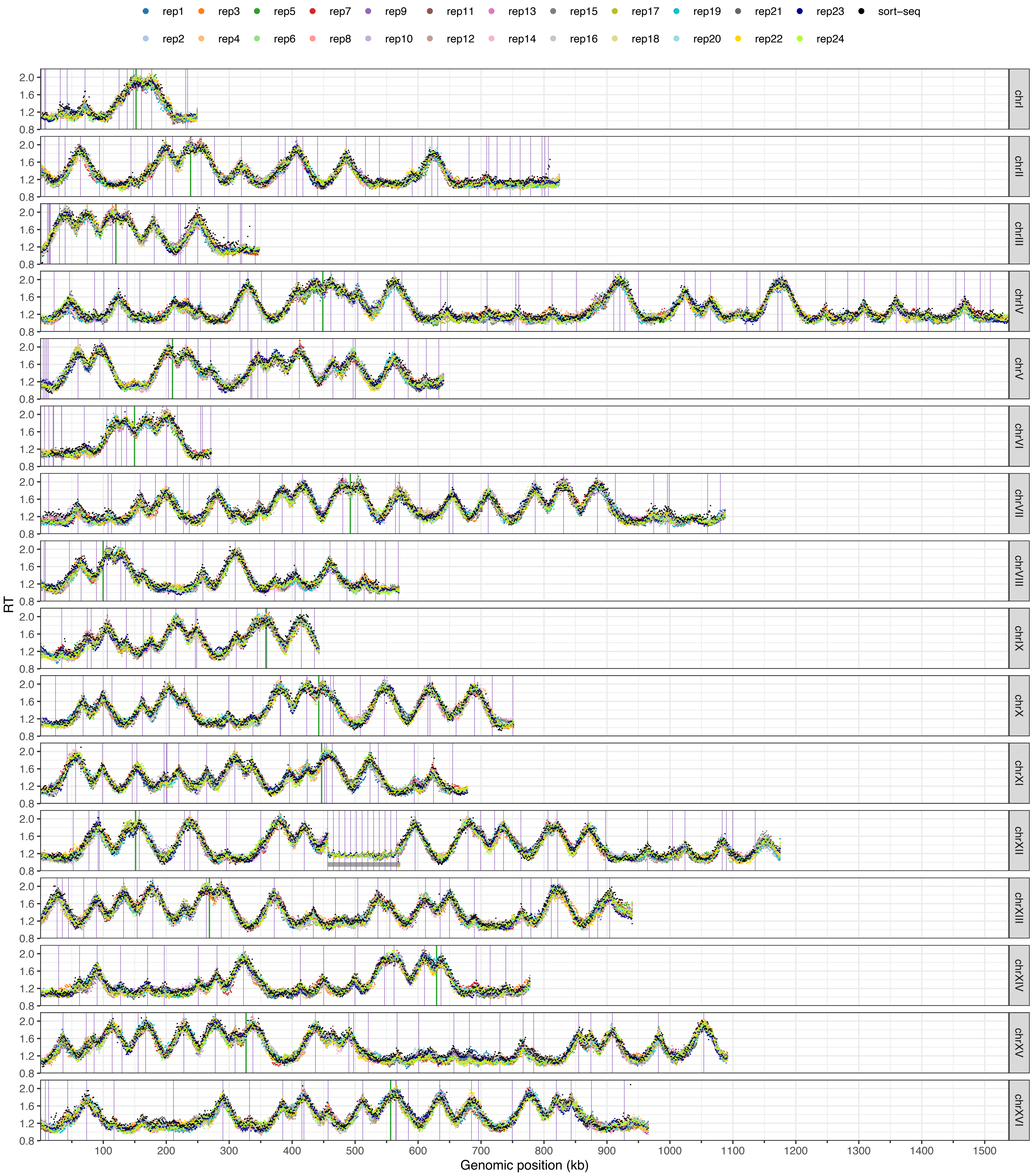

Supplementary Figure 24. Mean BrdU content profiles of all chromosomes of BT1 cells from a multiplexed PromethION run with 24 barcoded samples of BT1 BrdU-labelled DNA. The corresponding sort-seq relative copy number profile is also shown. See Fig. 2 caption for details.

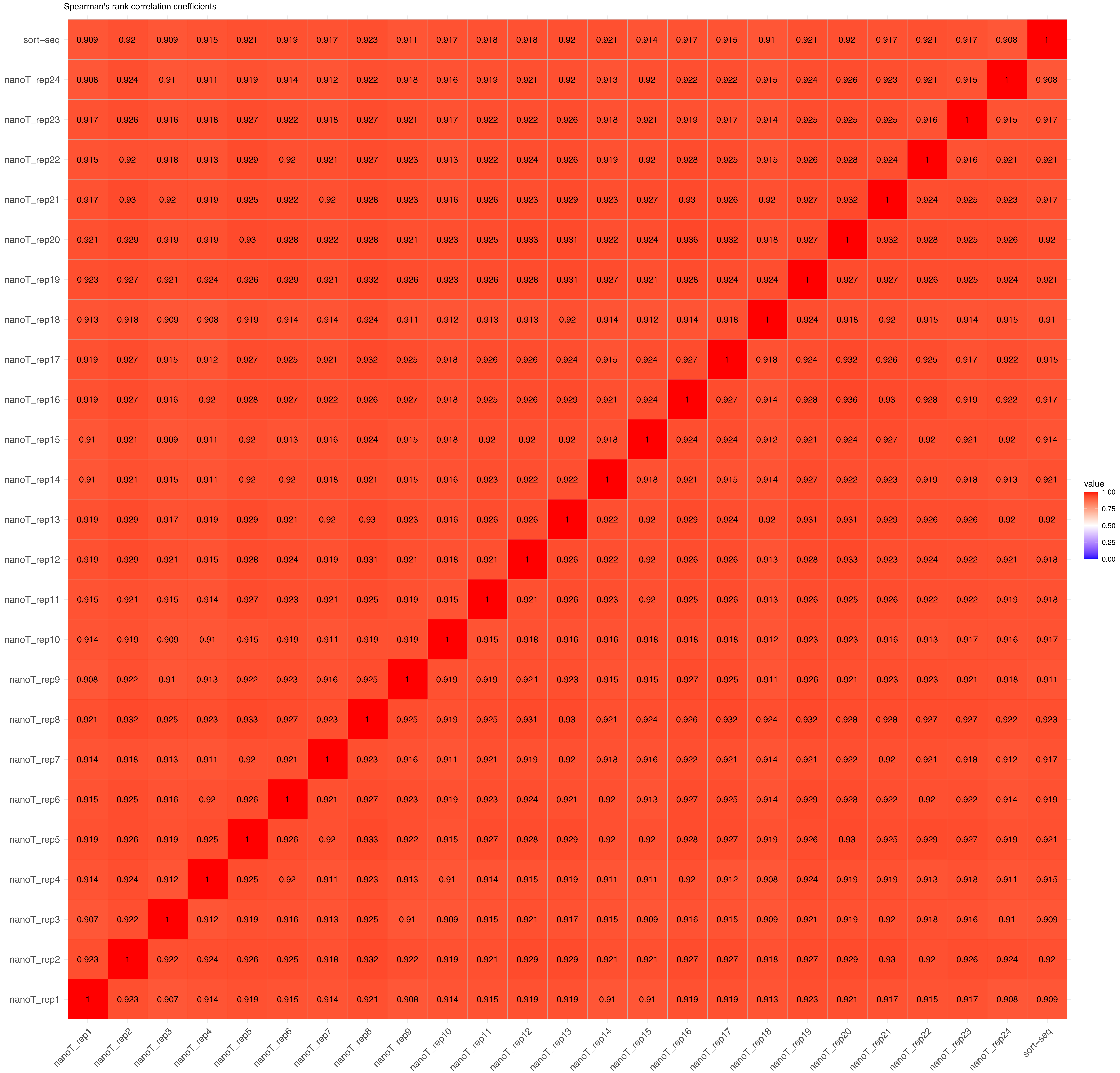

Supplementary Figure 25. Spearman's rank correlation coefficients of pairwise comparisons between mean BrdU content profiles of BT1 genome from a multiplexed PromethION run with 24 barcoded samples originating from the same BT1 BrdU-labelled genomic DNA. Comparison with BT1 genome sort-seq relative copy number profile is also provided. nanoT, Nanotiming; rep, replicate.

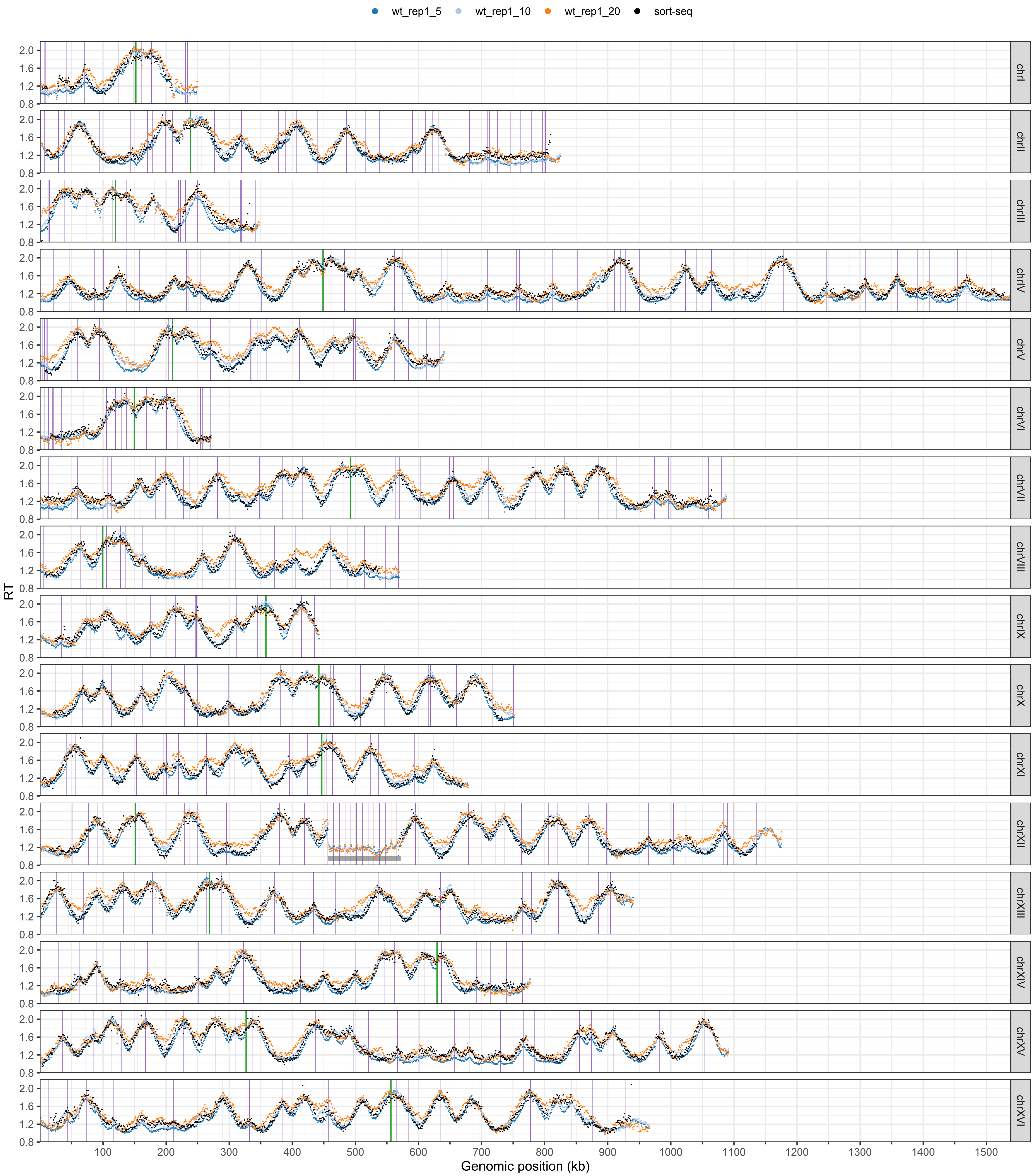

Supplementary Figure 26. Mean BrdU content profiles of *S. cerevisiae* chromosomes computed from reads of genomic DNA of BT1 cells labelled with 5, 10 or 20 μM BrdU. BT1 cells were labelled with BrdU for one doubling time. BT1 sort-seq relative copy number profile is shown for comparison. See Fig. 2 caption for details.

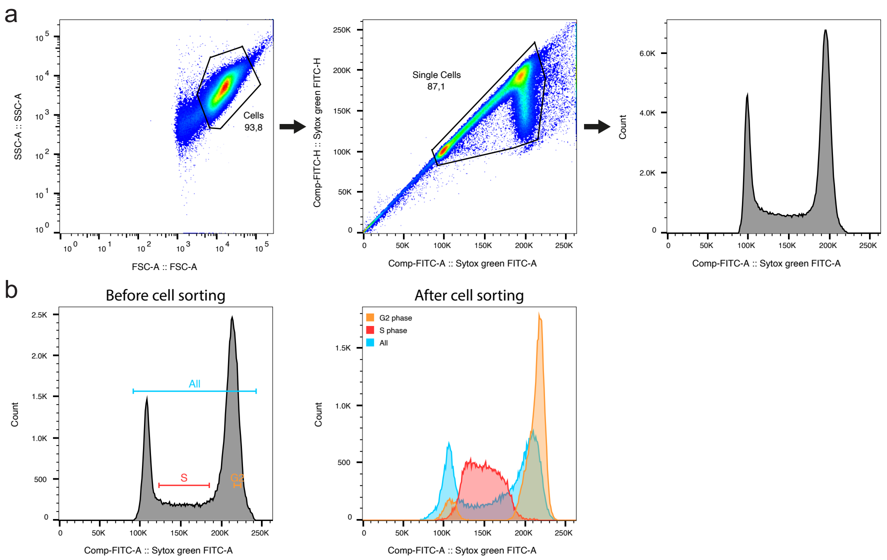

Supplementary Figure 27. Flow cytometry analysis. a, Gating strategy. Cells are fixed in ethanol and DNA is counterstained with SYTOX Green prior to flow cytometry analysis. Cells are initially gated using the FSC-Area versus SSC-Area plot to remove debris (left panel), then interrogated by the ratios of area (Sytox green FITC-A) to height (Sytox green FITC-H) of the SYTOX Green signal to gate out cell doublets (middle panel). A SYTOX Green area (Sytox green FITC-A) histogram shows DNA content after gating (right panel). **b**, Sorting of BT1 cells. Cells in S and G2 phases of the cell cycle, as well as an “All cells” control, were sorted using the indicated gates positioned on the DNA content histogram (left panel). Sorted cell populations are presented on the right panel.

	R9.4.1 chemistry	R10.4.1 chemistry			
	24 barcodes	No barcode	24 barcodes	48 barcodes	96 barcodes
PromethION flow cell price	$813	$600-900*	$600-900*	$600-900*	$600-900*
Library price	$529	$99	$99	$99	$99
Barcodes price	$312	-	$75	$150	$300
Total price	$1654	$699-999	$774-1074	$849-1149	$999-1299
Price per sample	$69	$699-999	$32-45	$18-24	$10-14

*Flow cell price depends on the quantity ordered.

Supplementary Table 1. Nanopore sequencing costs. Prices for R9.4.1 chemistry are those we paid in 2023/early 2024 (original prices in euros were converted into US dollars assuming an exchange rate of 1 USD/EUR); estimated flow cell, library and barcodes prices for R10.4.1 chemistry are from https://store.nanoporetech.com/promethion-flow-cell-packs-r10-4-1-m-version.html and https://store.nanoporetech.com/native-barcoding-kit-96-v14.html (as of July 2024).

Genome assembly	BUSCO score	Primary reads at chromosome ends			
		Mapped reads	Aligned length (bp)	Average aligned length (bp)	Average soft-clipped length (bp)
S288C_R64 (sacCer3)	99.3%	14696	446603214	30389	2730
W303 (Berlin et al, 2015)	99.2%	16334	513139873	31415	1364
W303 (Matheson et al, 2017)	99.2%	15095	472846294	31325	1172
BT1	99.3%	22712	745193724	32811	839

Supplementary Table 2. Evaluation of BT1 assembly. The BUSCO score evaluates genome assembly completeness. Assembly quality at chromosome ends was evaluated by extracting primary alignments overlapping telomeres and/or the 10 kb adjacent window (i.e., subtelomeric regions) followed by read number counting and estimation of mapped and soft-clipped lengths. See Methods.
