# Supplementary material for: Telomere-to-telomere DNA replication timing profiling using single-molecule sequencing with Nanotiming
Source: Nat Commun. 2025 Jan 2;16:242. doi: 10.1038/s41467-024-55520-3 (PMC11696806; doi:10.1038/s41467-024-55520-3)
Supplement: Supplementary file 2 — Description of Additional Supplementary Files [file 41467_2024_55520_MOESM2_ESM.pdf]

Description of Additional Supplementary Files

File Name: Supplementary Data 1

Description: Spearman's rank correlation coefficients of comparisons between telomere length and RT at the single-telomere level in wild-type, *rif1*Δ, *yku70*Δ, *ctf19*Δ and *fkh1*Δ BT1 cells. No data was computed at TEL13R in *yku70*Δ mutant because of a missing Y' element at the right end of chromosome XIII compared to BT1 assembly, preventing proper read mapping. Statistical significance was set to $p < 0.01$. n, number of measurements; rho, Spearman's rank correlation coefficient; RT, replication timing (mean BrdU content data were rescaled as in Fig. 2); wt, wild-type.

File Name: Supplementary Data 2

Description: Spearman's rank correlation coefficients of comparisons between telomere length and RT at the single-telomere level according to telomere X/XY' status in wild-type, *rif1*Δ, *yku70*Δ, *ctf19*Δ and *fkh1*Δ BT1 cells. See Supplementary Data 1 caption for details.

File Name: Supplementary Data 3

Description: Spearman's rank correlation coefficients of comparisons between telomere length and RT at the single-telomere level for each chromosome end in wild-type, *rif1*Δ, *yku70*Δ, *ctf19*Δ and *fkh1*Δ BT1 cells. See Supplementary Data 1 caption for details.

File Name: Supplementary Data 4

Description: Detailed sequencing information for Nanotiming samples. n_read_total, total number of reads used as input for BrdU basecalling; n_reads, number of mapped reads (mitochondrial reads were excluded); median read length was calculated for mapped reads.
