# Supplementary material for: Telomere-to-telomere DNA replication timing profiling using single-molecule sequencing with Nanotiming
Source: Nat Commun. 2025 Jan 2;16:242. doi: 10.1038/s41467-024-55520-3 (PMC11696806; doi:10.1038/s41467-024-55520-3)
Supplement: Supplementary file 7 — Reporting Summary [file 41467_2024_55520_MOESM7_ESM.pdf]

nature portfolio

Corresponding author(s): Benoît Le Tallec
Last updated by author(s): Nov 29, 2024

Reporting Summary

Nature Portfolio wishes to improve the reproducibility of the work that we publish. This form provides structure for consistency and transparency in reporting. For further information on Nature Portfolio policies, see our Editorial Policies and the Editorial Policy Checklist.

Statistics

For all statistical analyses, confirm that the following items are present in the figure legend, table legend, main text, or Methods section.

n/a	Confirmed	
☐	☒	The exact sample size (*n*) for each experimental group/condition, given as a discrete number and unit of measurement
☐	☒	A statement on whether measurements were taken from distinct samples or whether the same sample was measured repeatedly
☐	☒	The statistical test(s) used AND whether they are one- or two-sided<br>*Only common tests should be described solely by name; describe more complex techniques in the Methods section.*
☒	☐	A description of all covariates tested
☒	☐	A description of any assumptions or corrections, such as tests of normality and adjustment for multiple comparisons
☐	☒	A full description of the statistical parameters including central tendency (e.g. means) or other basic estimates (e.g. regression coefficient) AND variation (e.g. standard deviation) or associated estimates of uncertainty (e.g. confidence intervals)
☐	☒	For null hypothesis testing, the test statistic (e.g. *F*, *t*, *r*) with confidence intervals, effect sizes, degrees of freedom and *P* value noted<br>*Give P values as exact values whenever suitable.*
☒	☐	For Bayesian analysis, information on the choice of priors and Markov chain Monte Carlo settings
☒	☐	For hierarchical and complex designs, identification of the appropriate level for tests and full reporting of outcomes
☒	☐	Estimates of effect sizes (e.g. Cohen's *d*, Pearson's *r*), indicating how they were calculated

Our web collection on statistics for biologists contains articles on many of the points above.

Software and code

Policy information about availability of computer code

Data collection	MinKNOW (Oxford Nanopore Technologies, MinKNOW Core versions 4.5.4, 5.2.2, 5.4.3, 5.7.5 and 5.3.0-rc6-p2solo) for MinION and PromethION sequencing data acquisition; BD FACSChorus version 1.3.2 for flow cytometry data acquisition.
Data analysis	FlowJo version 10.9.0, Megalodon (Oxford Nanopore Technologies, versions 2.2.9 and 2.5.0), Guppy (Oxford Nanopore Technologies, versions 4.4.1 and 6.6.2), R version 4.0.5, minimap2 versions 2.24 and 2.26, Seqtk version 1.3, Rasusa version 0.7.0, Canu version 2.2, Racon version 1.5, Medaka version 1.7.2, Pilon version 1.23, Ragout version 2.3, LRSDAY version 1.7, Flye v2.9, bwa mem version 0.7.17-r1198-dirty, samtools versions 1.17 and 1.19, samtools view version 1.13, bedtools bamtobed version 2.26.0, rtracklayer R package version 1.62.0, bowtie version 2.4.4, bedtools version 2.30, picard version 2.7.3, Porechop_ABI (https://github.com/bonsai-team/Porechop_ABI), Telofinder (https://github.com/GillesFischerSorbonne/telofinder), FastXtend (https://www.genoscope.cns.fr/fastxtend/), Megalodon-based BrdU basecaller (https://github.com/LacroixLaurent/NanoForkSpeed), localMapper (https://github.com/DNAReplicationLab/localMapper/), Repliscope (https://github.com/DNAReplicationLab/Repliscope/). Custom scripts used in this study can be accessed at https://github.com/LacroixLaurent/NanoTiming and https://doi.org/10.5281/zenodo.14017724.

For manuscripts utilizing custom algorithms or software that are central to the research but not yet described in published literature, software must be made available to editors and reviewers. We strongly encourage code deposition in a community repository (e.g. GitHub). See the Nature Portfolio guidelines for submitting code & software for further information.

Data

Policy information about availability of data

All manuscripts must include a data availability statement. This statement should provide the following information, where applicable:

- Accession codes, unique identifiers, or web links for publicly available datasets
- A description of any restrictions on data availability
- For clinical datasets or third party data, please ensure that the statement adheres to our policy

Nanopore and Illumina sequencing data generated in this study have been deposited in the ENA database under accession code PRJEB76824 (https://www.ebi.ac.uk/ena/browser/view/PRJEB76824). Source data and BT1 assembly with genomic annotations are available at https://github.com/LacroixLaurent/NanoTiming and https://doi.org/10.5281/zenodo.14017724. S288C R64 (sacCer3) genome assembly (GCF_000146045.2) and W303 assemblies from Berlin et al, 2015 (GCA_000773925.1) and Matheson et al, 2017 (GCA_002163515.1) were downloaded from https://www.ncbi.nlm.nih.gov/datasets/genome/GCF_000146045.2/, https://www.ncbi.nlm.nih.gov/datasets/genome/GCA_000773925.1/ and https://www.ncbi.nlm.nih.gov/datasets/genome/GCA_002163515.1/, respectively. MCM869 nanopore sequencing data from Theulot et al, 2022 are accessible from ENA repository under accession code PRJEB50302 (https://www.ebi.ac.uk/ena/browser/view/PRJEB50302). ARS positions from OriDB (Siow et al, 2012) are available at http://cerevisiae.oridb.org/. MFA-seq data from Muller et al, 2014, sort-seq data for ctf19Δ cells from Natsume et al, 2013 and sort-seq data for rif1Δ cells from Hafner et al, 2018 are accessible from NCBI's Gene Expression Omnibus GEO repository under accession codes GSE48212 (https://www.ncbi.nlm.nih.gov/geo/query/acc.cgi?acc=GSE48212, GSM1180746 and GSM1180747 samples), GSE41982 (https://www.ncbi.nlm.nih.gov/geo/query/acc.cgi?acc=GSE41982, GSM1029480 and GSM1029481 samples) and GSE97953 (https://www.ncbi.nlm.nih.gov/geo/query/acc.cgi?acc=GSE97953, GSM2583609 and GSM2583613 samples), respectively.

Research involving human participants, their data, or biological material

Policy information about studies with human participants or human data. See also policy information about sex, gender (identity/presentation), and sexual orientation and race, ethnicity and racism.

Reporting on sex and gender	Not applicable.
Reporting on race, ethnicity, or other socially relevant groupings	Not applicable.
Population characteristics	Not applicable.
Recruitment	Not applicable.
Ethics oversight	Not applicable.

Note that full information on the approval of the study protocol must also be provided in the manuscript.

Field-specific reporting

Please select the one below that is the best fit for your research. If you are not sure, read the appropriate sections before making your selection.

☒ Life sciences ☐ Behavioural & social sciences ☐ Ecological, evolutionary & environmental sciences

For a reference copy of the document with all sections, see nature.com/documents/nr-reporting-summary-flat.pdf

Life sciences study design

All studies must disclose on these points even when the disclosure is negative.

Sample size	No sample size calculation was performed. For replication timing profiles, sample sizes range from a minimum of 3 to 6 for each strain, with the rationale that every strain is analysed independently at least three times. For telomere studies, sample sizes correspond to the number of experiments (3 to 6) multiplied by the number of telomeres (32 for all strains except yku70, for which only 31 telomeres could be analysed). Finally, 7,000 to 25,000 individual telomeres were scored to analyse the relationship between telomere length and replication timing; these sample sizes were deemed sufficient to perform robust analyses.
Data exclusions	Telomere TEL13R was not analysed in yku70Δ mutant due to the loss of a Y' element at the right end of chromosome XIII compared to BT1 assembly, preventing proper read mapping; it was therefore removed and excluded from our analyses.
Replication	To verify the reproducibility of our experimental findings, Nanotiming profiling of the replication timing (RT) program of the yeast genome was performed independently at least 3 times in a wild-type and in 4 different mutant strains, with all biological replicates giving comparable results for a given strain. Those replicates were also used to compute RT at individual telomeres as well as telomere length, once again giving similar results. The flow cytometry experiment analysing BrdU labelling impact on BT1 cell cycle was performed three times independently with similar results. The sort-seq experiment used to generate a control replication timing profile was performed only once since it accurately recapitulated published sort-seq profiles of wild-type yeast strains. The pilot experiment testing different BrdU concentrations, which was merely exploratory work, was also performed once.

Randomization | Randomization is not applicable to our study. Data shown herein are genome-wide replication timing profiles of S. cerevisiae genome or individual telomere lenght or replication timing in wild-type or mutant strains. Samples therefore correspond per se to wild-type or mutant cells or to specific features and need not be further allocated into experimental groups.

Blinding | Blinding is not applicable to our study. As specified above, there was no group allocation. Data shown herein are genome-wide replication timing profiles of S. cerevisiae genome or individual telomere lenght or replication timing in wild-type or mutant strains, which were analysed with the same parameters by an automated pipeline, "blind" by definition to samples' identity.

Reporting for specific materials, systems and methods

We require information from authors about some types of materials, experimental systems and methods used in many studies. Here, indicate whether each material, system or method listed is relevant to your study. If you are not sure if a list item applies to your research, read the appropriate section before selecting a response.

Materials & experimental systems

n/a	Involved in the study
☒	☐ Antibodies
☒	☐ Eukaryotic cell lines
☒	☐ Palaeontology and archaeology
☒	☐ Animals and other organisms
☒	☐ Clinical data
☒	☐ Dual use research of concern
☒	☐ Plants

Methods

n/a	Involved in the study
☒	☐ ChIP-seq
☐	☒ Flow cytometry
☒	☐ MRI-based neuroimaging

Plants

Seed stocks | Not applicable.

Novel plant genotypes | Not applicable.

Authentication | Not applicable.

Flow Cytometry

Plots

Confirm that:

☒ The axis labels state the marker and fluorochrome used (e.g. CD4-FITC).

☒ The axis scales are clearly visible. Include numbers along axes only for bottom left plot of group (a 'group' is an analysis of identical markers).

☐ All plots are contour plots with outliers or pseudocolor plots.

☐ A numerical value for number of cells or percentage (with statistics) is provided.

Methodology

Sample preparation | S. cerevisiae cells were fixed in 70% ethanol, washed with 50 mM sodium citrate pH 7.4, incubated for 1 hour at 50°C in sodium citrate buffer supplemented with 0.25 mg.ml-1 RNAse A, added with 2 mg.ml-1 proteinase K and incubated for one additional hour. DNA was counterstained overnight with 1 µM SYTOX Green.

Instrument | Cells were analysed and sorted using a BD FACSMelody™ Cell Sorter.

Software | Data were collected using BD FACSChorus version 1.3.2 and analysed using FlowJo version 10.9.0.

Cell population abundance | Cells in S and G2 phases of the cell cycle, as well as an “All cells” control, were sorted using gates positioned on the DNA content (Sytox green FITC-Area) histogram. To determine the purity of sorted fractions, post-sort cells were directly re-analyzed by flow cytometry. Sorting results are presented in the Supplementary Information.

Gating strategy

Cells previously fixed in ethanol with DNA counterstained with SYTOX Green were initially gated using the FSC-Area versus SSC-Area plot to remove debris, then interrogated by the ratios of area (Sytox green FITC-A) to height (Sytox green FITC-H) of the SYTOX Green signal to gate out cell doublets.

☒ Tick this box to confirm that a figure exemplifying the gating strategy is provided in the Supplementary Information.
